# Asymmetric total synthesis of tricyclic prostaglandin D2 metabolite methyl ester via oxidative radical cyclization

**DOI:** 10.3762/bjoc.21.152

**Published:** 2025-09-24

**Authors:** Miao Xiao, Liuyang Pu, Qiaoli Shang, Lei Zhu, Jun Huang

**Affiliations:** 1 School of Chemistry and Chemical Engineering, University of South China, Hengyang 421001, China; 2 College of Pharmacy, Third Military Medical University, Chongqing 200038, China

**Keywords:** asymmetric total synthesis, oxidative radical cyclization, tricyclic prostaglandin D_2_ metabolite methyl ester

## Abstract

Prostaglandin D_2_ (PGD_2_) is a key pathophysiological mediator in many human diseases and biological pathways. Tricyclic prostaglandin D_2_ metabolite methyl ester (tricyclic-PGDM methyl ester), the major urinary metabolite of PGD_2_, can be used as a clinical indicator for PGD_2_ overproduction. However, the limited amount of tricyclic-PGDM methyl ester available has prevented its practical use, and synthesis methods for tricyclic-PGDM methyl ester are required. Based on the utilization of oxidative radical cyclization for the stereoselective construction of the cyclopentanol subunit with three consecutive stereocenters, we describe an asymmetric total synthesis of tricyclic-PGDM methyl ester in 9 steps and 8% overall yield.

## Introduction

Prostaglandins (PGs), a family of hormone-like lipid compounds, are ubiquitous natural products that control many essential biological processes in animals and humans [1–4]. In particular, prostaglandin D_2_ (PGD_2_, **3**) is a key pathophysiological mediator in a number of human diseases and biological pathways, such as systemic mastocytosis and inflammation. Therefore, the development of methods for sensitive detection of endogenous PGD_2_ production and its stereoisomers are clinically important [5]. However, PGD_2_ is rapidly metabolized with a short half-life, making the identification and quantification of its downstream metabolites a promising and reliable diagnostic tool. Tricyclic prostaglandin D_2_ metabolite methyl ester (tricyclic-PGDM methyl ester, **4**), the major urinary metabolite of PGD_2_, has been used as an indicator for PGD_2_ overproduction. Roberts and associates established an assay for tricyclic-PGDM measurement using ^18^O-labelled tricyclic-PGDM methyl ester **8**, which is an effective tool in clinical applications [6]. However, the scarcity of metabolite **4** has prevented it from being used more widely, and thus synthesis methods for **4** are required.

**Scheme 1:**
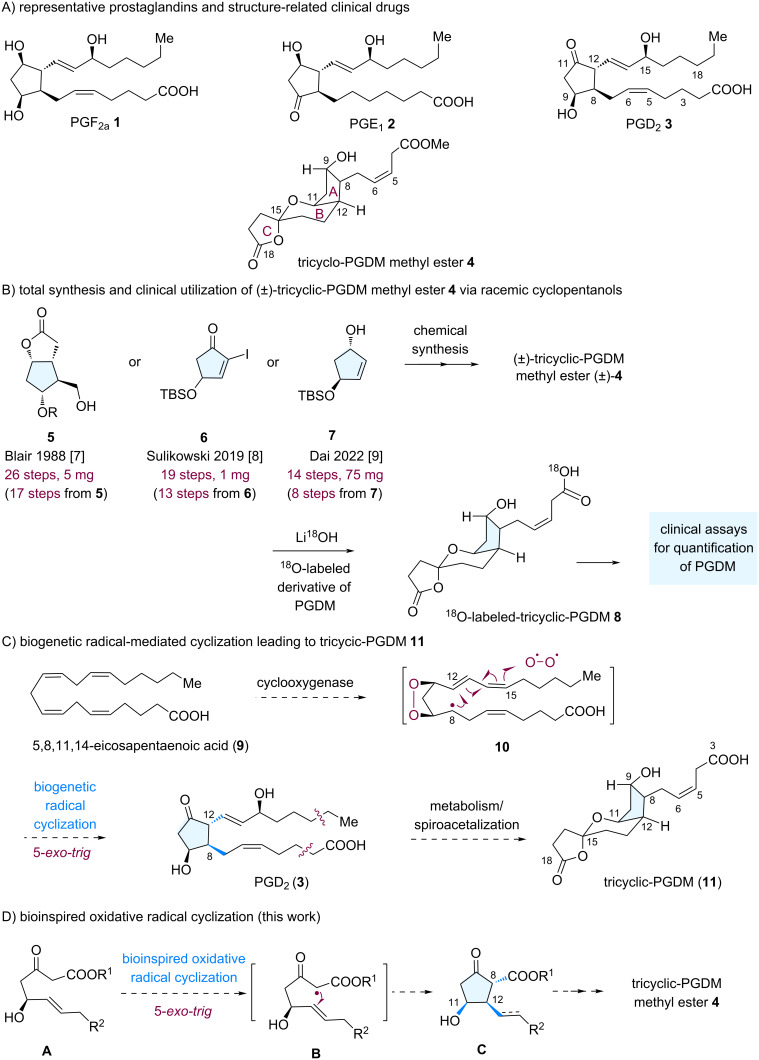
Representative prostaglandins and general synthetic strategy toward PGDM methyl ester **4**.

Compound **4** contains a cyclopentanol scaffold with stereogenicity at C8, C9, C11, and C12 (Scheme 1A). In addition, **4** is synthetically challenging because of the tricyclic ring system, spiroketal moiety (B, C-ring), and instability arising from dehydration of the hydroxy groups. Prior approaches to (±)-**4** have shown the feasibility of accessing this target molecule. Blair [7] and Sulikowski [8] reported the total synthesis of (±)-**4** from pentacyclic starting materials **5** and **6**, respectively (Scheme 1B). In 2021, Dai reported the total synthesis of (±)-**4** from cyclopentanol **7**, in which the bicyclic spiroketal moiety and (*Z*)-3-butenoate side chain were formed via a palladium-catalyzed carbonylative spirolactonization and *Z*-selective cross-metathesis, respectively [9]. In general, racemic cyclopentanol precursors (A ring system, prepared in 6–9 steps) have been used to form the polyfunctionalized tricyclic frameworks incorporating contiguous stereocenters.

In previous syntheses, the efficient construction of the cyclopentanol ring system with the appropriate functional groups in place for attaching the remaining groups is a highly important task for the asymmetric total synthesis of PGs and analogues [10–13]. The groups of Aggarwal [14], Hayashi [15], and Zhang [16] have reported bond-disconnection strategies for the total syntheses of PGs via organocatalysis, and enyne cycloisomerization, respectively. Thus, from a strategic viewpoint, developing alternative synthetic approaches for the stereoselective construction of the highly substituted cyclopentanol core framework in compound **4** may advance the efficient total synthesis of **4** and is required to explore alternative synthetic strategies for PGs and analogues [17].

Biosynthetically, **4** is proposed to arise via a 5-*exo*-*trig* biogenetic radical-mediated cyclization (Scheme 1C) [18–19]. Over the past five decades, the Snider oxidative radical reaction has been used as a powerful method for synthesizing complex natural products [20–21]. We envisaged that the A-ring in **4** could be constructed from the alkene-substituted β-keto ester precursor **A** via a bioinspired oxidative radical cyclization (Scheme 1D).

Herein, we report the full details of our efforts to stereoselectively access the *syn*-*anti*-cyclopentanol ring system with three vicinal stereogenic centers at C11, C12, and C8 via the oxidative radical cyclization that led to the asymmetric total synthesis of compound **4**.

## Results and Discussion

### First generation asymmetric total synthesis of tricyclic-PGDM methyl ester

**Scheme 2:**
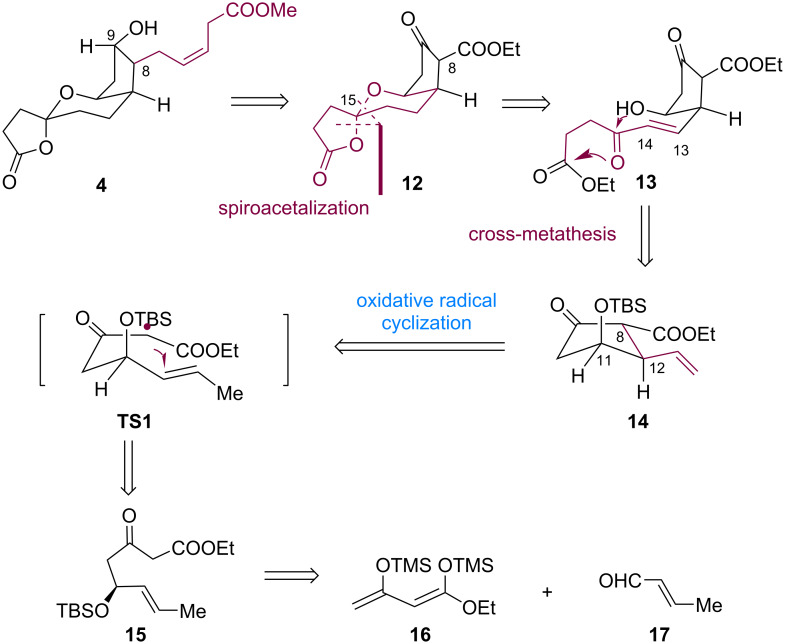
Retrosynthetic analysis for the first generation synthesis of PGDM methyl ester **4**.

The retrosynthetic analysis of tricyclic-PGDM methyl ester **4** is shown in Scheme 2. We expected to derive **4** from tricyclic substrate **12** via a side-chain installation at C8 [22]. The spiroketal moiety in compound **12** could be obtained from compound **13** via a diastereoselective spiroketalization dictated by the anomeric effect [23] in the tricyclic scaffold. Compound **13** could be produced from olefin **14** via cross-metathesis. The regio- and diastereoselective connection of C8 and C12 in compound **14** could be realized through a transition-metal-mediated oxidative radical cyclization through **TS-1** from β-keto ester **15** [24]. The β-keto ester **15** was expected to be derived from compounds **16** and **17** via an asymmetric aldol reaction [25].

**Scheme 3:**
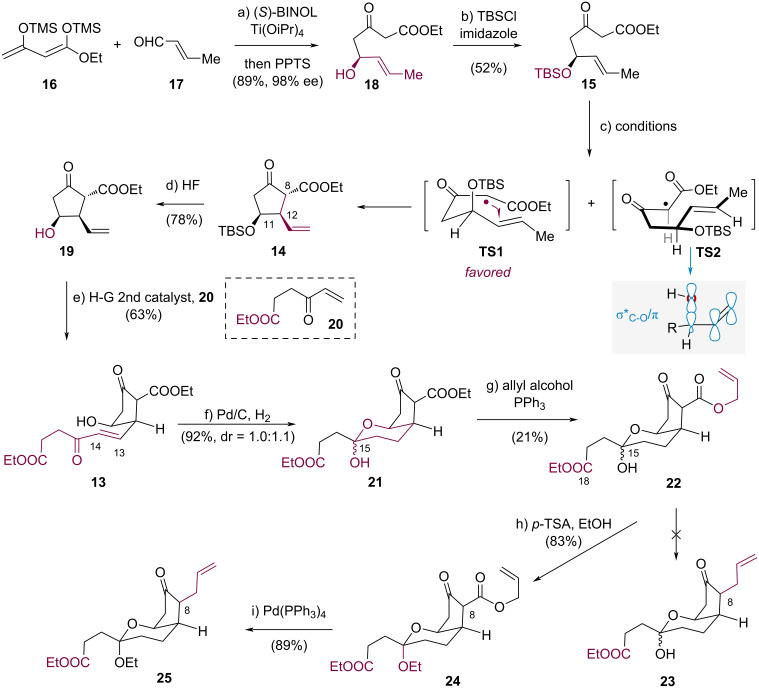
Synthesis of bicyclic ketal **25**.

The first phase of the synthesis required the efficient preparation of compound **14**, for which a transition-metal-mediated oxidative radical cyclization of β-keto ester **15** was initially investigated (Scheme 3). Chan’s diene (**16**) was subjected to condensation with freshly distilled aldehyde **17** in THF at room temperature, using a catalytic system comprising Ti(OiPr)_4_/(*S*)-BINOL complex (2.0 mol %). Subsequent deprotection with pyridinium *p*-toluenesulfonate (PPTS) at 0 °C afforded the corresponding alcohol **18** in 89% yield with excellent enantioselectivity (98% ee) [25]. The hydroxy group in **18** was then protected via treatment with TBSCl in the presence of Et_3_N in CH_2_Cl_2_, yielding β-keto ester **15** in 52% yield.

With diketone **15** in hand, we subsequently investigated the transition-metal-mediated oxidative radical cyclization for constructing cyclopentanone **14**. First, we used Mn(OAc)_3_·2H_2_O/Cu(OAc)_2_·H_2_O [21] as the oxidant system to perform oxidative annulation of β-keto ester **15** in MeCN as solvent. However, only 9% of the desired product **14** was obtained after conducting the reaction at 50 °C for 36 h, and extensive decomposition of the starting material β-keto ester **15** occurred (Table 1, entry 1). Solvent screening of EtOH [26], acetic acid [26], and hexafluoroisopropanol (HFIP) [27] demonstrated that HFIP afforded optimal results, delivering cyclopentanone **14** in 63% yield as a single diastereomer (Table 1, entry 4). To explain this diastereoselectivity, we hypothesize that the C–O bond, which occupies an axial position in the proposed transition state **TS-1**, could avert an additional hyperconjugative interaction (σ*_C-O_/π) that renders the reacting C=C bond electron-deficient [28], thereby lowering the energy barrier for electrophilic radical addition. Increasing the reaction temperature to 70 °C proved detrimental, yielding only trace amounts of product **14** (Table 1, entry 5). Finally, replacing Mn(OAc)_3_·2H_2_O/Cu(OAc)_2_·H_2_O with other oxidants, such as ceric ammonium nitrate (CAN) [29–30] and Fe(ClO_4_)_3_·9H_2_O [31], failed to afford desired product **14** (Table 1, entries 6 and 7).

**Table 1:** Optimization of conditions to convert diketone **15** into cyclopentanone **14**.^a^

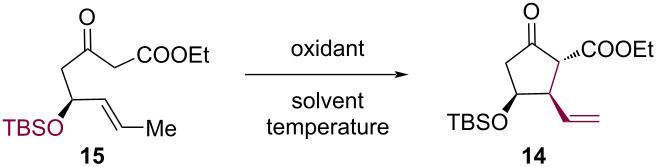				

entry	oxidant	solvent	temp. (°C)	yield (%)

1	Mn(OAc)_3_·2H_2_O (2.2 equiv), Cu(OAc)_2_·H_2_O (1.1 equiv)	MeCN	50	9
2	Mn(OAc)_3_·2H_2_O (2.2 equiv), Cu(OAc)_2_·H_2_O (1.1 equiv)	EtOH	50	0
3	Mn(OAc)_3_·2H_2_O (2.2 equiv), Cu(OAc)_2_·H_2_O (1.1 equiv)	AcOH	50	12
4	Mn(OAc)_3_·2H_2_O (2.2 equiv), Cu(OAc)_2_·H_2_O (1.1 equiv)	HFIP	50	63
5	Mn(OAc)_3_·2H_2_O (2.2 equiv), Cu(OAc)_2_·H_2_O (1.1 equiv)	HFIP	70	trace
6	Fe(ClO_4_)_3_·9H_2_O (2.2 equiv)	HFIP	50	0
7	CAN (2.2 equiv)	HFIP	50	0

^a^Step c in Scheme 3.

To explore the synthesis of fully functionalized tricyclic core scaffold **12**, methods for incorporating the side chain at C14 and for the stereocontrolled introduction of the allyl moiety at C8 were developed. A straightforward transformation was designed involving a cross-metathesis of the C13–C14 double bond and a palladium-catalyzed decarboxylative allylation [32] as the key steps.

With **14** in hand, we investigated the feasibility of cross-metathesis of the C13–C14 double bond. Initially, compounds **14** and **20** were evaluated for the cross-metathesis of the olefin moiety in **14**. No reaction occurred and the desired product was not detected (not shown), presumably because of the steric hindrance from the TBS group. Following the removal of the silyl group, cyclopentanol **19** underwent the cross-metathesis reaction smoothly in the presence of the Hoveyda–Grubbs second-generation catalyst to afford the enone **13** in 63% yield with the desired *trans*-configuration. Enone **13** was then subjected to the Pd/C-catalyzed hydrogenation to give the thermodynamically favored bicyclic hemiketal **21** in 92% yield as an inseparable mixture of diastereomers at C-15 in a ratio of 1.0/1.1 (^1^H NMR analysis).

Having established a route to the bicyclic hemiketal **21**, we investigated the stereoselective introduction of an allyl moiety at C8 for the synthesis of compound **25** according to the strategy in Scheme 3. Treatment of **21** with allyl alcohol and triphenylphosphine afforded transesterification product **22** in 21% yield [33], accompanied by unidentified decarboxylation by-products. A variety of standard conditions failed to promote the palladium-catalyzed decarboxylative allylation of allylic β-ketocarboxylate intermediate **22** (see Supporting Information File 1 for the details). Reasoning that the preferential coordination of the palladium catalyst with the hydroxy group at C15 and the carbonyl group at C18 in compound **22** may have deactivated the palladium catalyst [34], we protected the hydroxy group. Compound **22** was treated with *p*-toluenesulfonic acid (*p*-TSA) in EtOH at room temperature to afford ketal **24** in 83% yield as a single diastereomer. Subsequently, palladium-catalyzed decarboxylative allylation delivered compound **25** in 89% yield.

The efficiency of our first-generation strategy for asymmetric synthesis of **4** was unsatisfactory because it required nine steps to prepare bicyclic intermediate **25** with an overall yield of just 2.0%. This low efficiency prompted us to develop a more streamlined synthetic route for target compound **4**.

### Second generation asymmetric total synthesis of tricyclic-PGDM methyl ester

Although the efficiency of the first-generation asymmetric total synthesis strategy was limited, the development of synthetic methods during this work, particularly the transition-metal-mediated oxidative radical cyclization for stereoselective assembly of the cyclopentanol scaffold bearing the C8, C11, and C12 contiguous stereogenic centers, provided important insights that influenced the design of our second-generation total synthesis. Compared with the Snider-type radical cyclization using stoichiometric amounts of metal oxidants, visible-light-induced photoredox-catalyzed radical cyclization strategies have emerged as an effective synthetic route for the stereocontrolled construction of diverse, highly functionalized bioactive and pharmaceutical molecules [35–37].

**Scheme 4:**
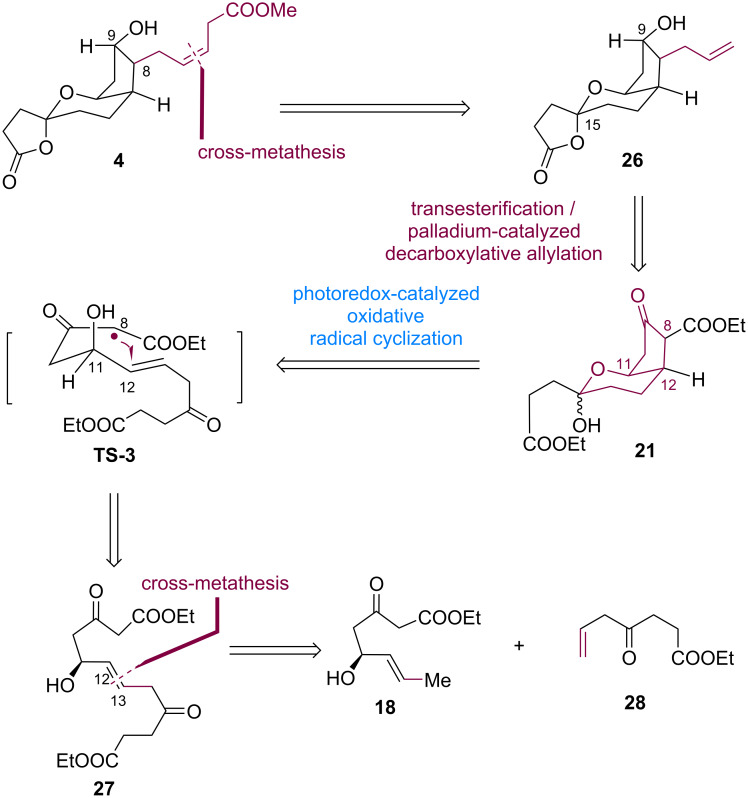
Retrosynthetic analysis for the second-generation synthesis of tricyclic PGDM methyl ester **4**.

The herein adopted synthetic strategy, employing photoredox-catalyzed radical cyclization, is illustrated in Scheme 4. Compound **4** was expected to be derived from tricyclic substrate **26** via a *Z*-selective cross-metathesis [9]. The allyl group in compound **26** could be installed in β-keto ester **21** via sequential transesterification [33] and palladium-catalyzed decarboxylative allylation [32]. The regio- and diastereoselective connection of C8 and C12 in compound **21** could be realized through a photoredox-catalyzed radical cyclization of unactivated alkene-substituted β*-*ketoester **27**. This reaction was expected to involve a 5-*exo*-*trig* radical cyclization via transition state **TS-3** [38], in which the diastereoselectivity could be controlled by the stereoelectronic effect of the axial hydroxy group at C11 (Scheme 4) [28].

**Scheme 5:**
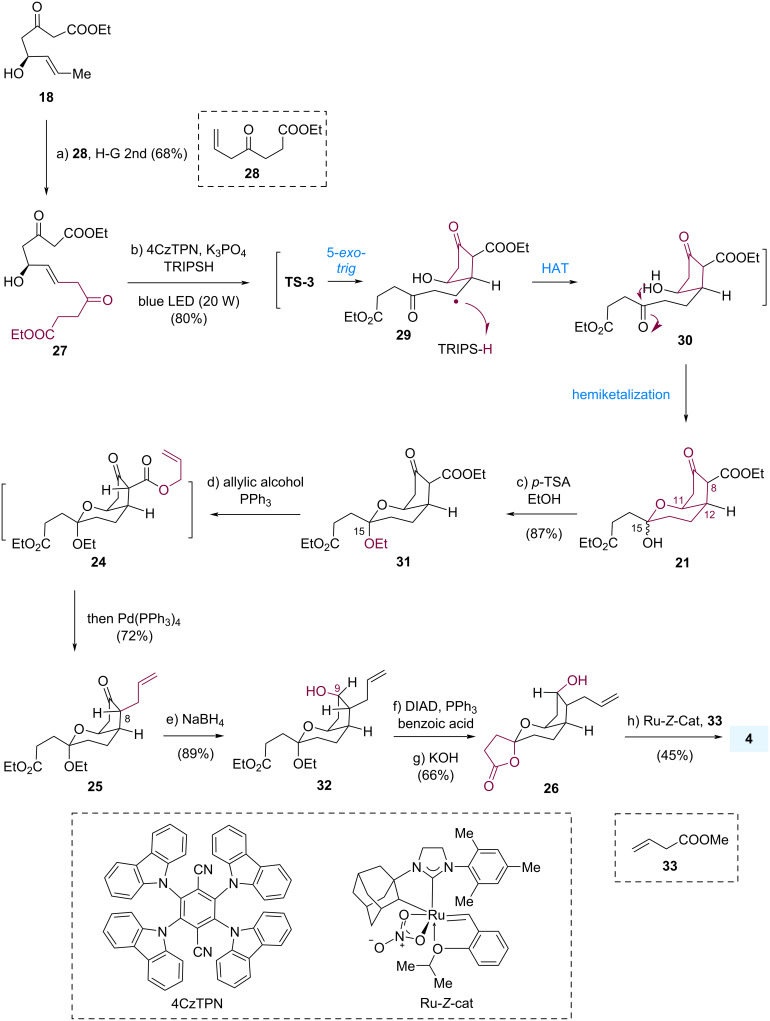
Asymmetric total synthesis of tricyclic-PGDM methyl ester **4**.

First, β-keto ester **21** was synthesized (Scheme 5). Cross-metathesis of allylic alcohol **18** and olefin **28** with the assistance of the Hoveyda–Grubbs second-generation catalyst delivered the desired product **27** in 68% yield. Having accessed the β-keto ester **27**, the photoredox-catalyzed oxidative radical cyclization of compound **27** was established on a 0.8 g scale, yielding compound **21** in 80% yield, as an inseparable mixture of diastereomers at C-15, the precursor for palladium-catalyzed decarboxylative allylation. The proposed mechanism to **21** involved the formation of an electron-deficient, resonance-stabilized radical species, followed by intramolecular alkylation of the unactivated alkene to generate radical **29** via a diastereoselective 5-*exo-trig* cyclization step. Radical intermediate **29** was trapped by 2,4,6-triisopropylbenzenethiol (TRIPSH) through a hydrogen atom transfer (HAT) process to afford intermediate **30** [39], which then cyclized to yield product **21**.

To install the allyl group at C8 with the desired stereochemistry, we treated compound **21** with *p*-TSA in EtOH at room temperature, and ketal **31** was obtained in 87% yield as a single diastereomer. Subsequently, one-pot transesterification and palladium-catalyzed decarboxylative allylation delivered compound **25** in 72% yield.

Next, we expected that we could perform a chemo- and diastereoselective reduction of the ketone to introduce the hydroxy group at C9 in a single step. However, the diastereoselective reduction of the ketone in **25** was challenging because the ketone was embedded in the concave face, which was more sterically hindered than the convex face. Common reductants, such as NaBH_4_, DIBAL-H, and LiAlH(O*t*-Bu)_3_, provided product **32** with the opposite stereochemistry at C9. Therefore, alcohol **32** was subjected to a one-pot Mitsunobu reaction, hydrolyzation, and spirolactonization to give the corresponding alcohol **26** with inversed configuration at C9. Finally, terminal alkene **26** was transformed by Dai’s Ru-catalyzed *Z*-selective cross-metathesis with **33** [9,40] to provide compound **4** in 8% overall yield over 9 steps starting from the readily available compound **16** [41].

## Conclusion

In conclusion, we developed a synthetic strategy using a radical C_sp3_–H cyclization, including Snider oxidative radical cyclization and photoredox-catalyzed radical cyclization, to construct cyclopentanols with three contiguous stereogenic centers in compound **4**. Our total synthesis also features an efficient cross-metathesis reaction to produce photoredox-catalyzed radical cyclization reaction precursor **27**, a one-pot transesterification and palladium-catalyzed decarboxylative allylation to install the side chain at C8, and a diastereoselective spirolactonization to generate the spiroketal moiety in **26**. This total synthesis is promising for divergent total syntheses of other PGs and structurally related pharmaceutical derivatives.

## Supporting Information

File 1 Experimental procedures, characterization data and copies of ^1^H and ^13^C NMR spectra.

## Data Availability

All data that supports the findings of this study is available in the published article and/or the supporting information of this article.
