# Supplementary material for: Asymmetric total synthesis of tricyclic prostaglandin D2 metabolite methyl ester via oxidative radical cyclization
Source: Beilstein J Org Chem. 2025 Sep 24;21:1964–72. doi: 10.3762/bjoc.21.152 (PMC12477901; doi:10.3762/bjoc.21.152)
Supplement: File 1 — Experimental procedures, characterization data and copies of 1H and 13C NMR spectra. [file Beilstein_J_Org_Chem-21-1964-s001.pdf]

BEILSTEIN JOURNAL OF ORGANIC CHEMISTRY

Supporting Information

for

Asymmetric total synthesis of tricyclic prostaglandin D2 metabolite methyl ester via oxidative radical cyclization

Miao Xiao, Liuyang Pu, Qiaoli Shang, Lei Zhu and Jun Huang

Beilstein J. Org. Chem. **2025,** *21,* 1964–1972. doi:10.3762/bjoc.21.152

Experimental procedures, characterization data and copies of ^{1}H and ^{13}C NMR spectra

License and Terms: This is a supporting information file under the terms of the Creative Commons Attribution License (https://creativecommons.org/licenses/by/4.0). Please note that the reuse, redistribution and reproduction in particular requires that the author(s) and source are credited and that individual graphics may be subject to special legal provisions.
The license is subject to the *Beilstein Journal of Organic Chemistry* terms and conditions: (https://www.beilstein-journals.org/bjoc/terms)

Table of contents

1. Methods..S2
2. First generation asymmetric total synthesis of tricyclic-PGDM methyl ester..S3
Preparation of compound **18**:..S4
HPLC spectra of compound **18**:..S5
Preparation of compound **15**:..S6
Table S1. Optimization of reaction conditions..S7
Preparation of compound **14**:..S7
Preparation of compound **19**:..S8
Preparation of compound **20**:..S9
Preparation of compound **13**:..S10
Preparation of compound **21**:..S10
Preparation of compound **22**:..S11
Preparation of compound **24**:..S12
Preparation of compound **25**:..S13
3. Second generation asymmetric total synthesis of tricyclic-PGDM methyl ester..S14
Preparation of compound **28**:..S15
Preparation of compound **27**:..S15
Preparation of compound **21**:..S16
Preparation of compound **31**:..S16
Preparation of compound **25**:..S19
Preparation of compound **32**:..S19
Preparation of compound **26**:..S20
Preparation of compound **4**:..S21
4. NMR spectra for the synthesized compounds..S23

1. Methods

Unless otherwise noted, all reactions were carried out under an argon atmosphere with dry solvents. Reagents were purchased at the highest commercial quality and used without further purification. Solvents purification was conducted according to purification of laboratory vhemicals (Peerrin, D. D.; Armarego, W. L. and Perrins, D. R., Pergamon Press: Oxford, 1980). The reactions that require heating were maintained in an oil bath. Yields refer to chromatographically and spectroscopically (^{1}H NMR) homogeneous materials. Reactions were monitored by thin-layer chromatography (TLC) carried out on 0.25 mm Tsingdao silica gel plates (GF-254) using UV light at 254 nm as visualizing agent and ethanolic solution of phosphomolybdic acid (PMA) and cerium sulfate or basic aqueous potassium permanganate ($KMnO_4$) solution as staining agent. Tsingdao silica gel (60, particle size 0.040–0.063 mm) was neutralized with pH 7.0 buffer for flash column chromatography. NMR spectra were recorded on a Bruker Advance 500 (^{1}H: 500 MHz, ^{13}C: 125 MHz) and were calibrated using residual undeuterated solvent as an internal reference ($CDCl_3$: ^{1}H NMR = 7.26 ppm, ^{13}C NMR = 77.16 ppm). The following abbreviations were used to designate multiplicities: s = singlet, d = doublet, t = triplet, q = quartet, m = multiplet, br = broad. Optical rotations were determined on a JASCO P-1030 Polarimeter in the solvent indicated. IR spectra were recorded on an IR Prestige-21 FTIR spectrometer as KBr disc. High resolution mass spectrometry (HRMS) data were recorded on a Bruker Apex IV RTMS instrument and a VG Auto Spec-3000 spectrometer, respectively. For all HRMS measurements, the ionization method is ESI or APCI and the mass analyzer type is TOF. The ee values of compounds were determined by chiral HPLC (Shimadzu LC-16, CHIRALPAK AD-H column).

2. First generation asymmetric total synthesis of tricyclic-PGDM methyl ester

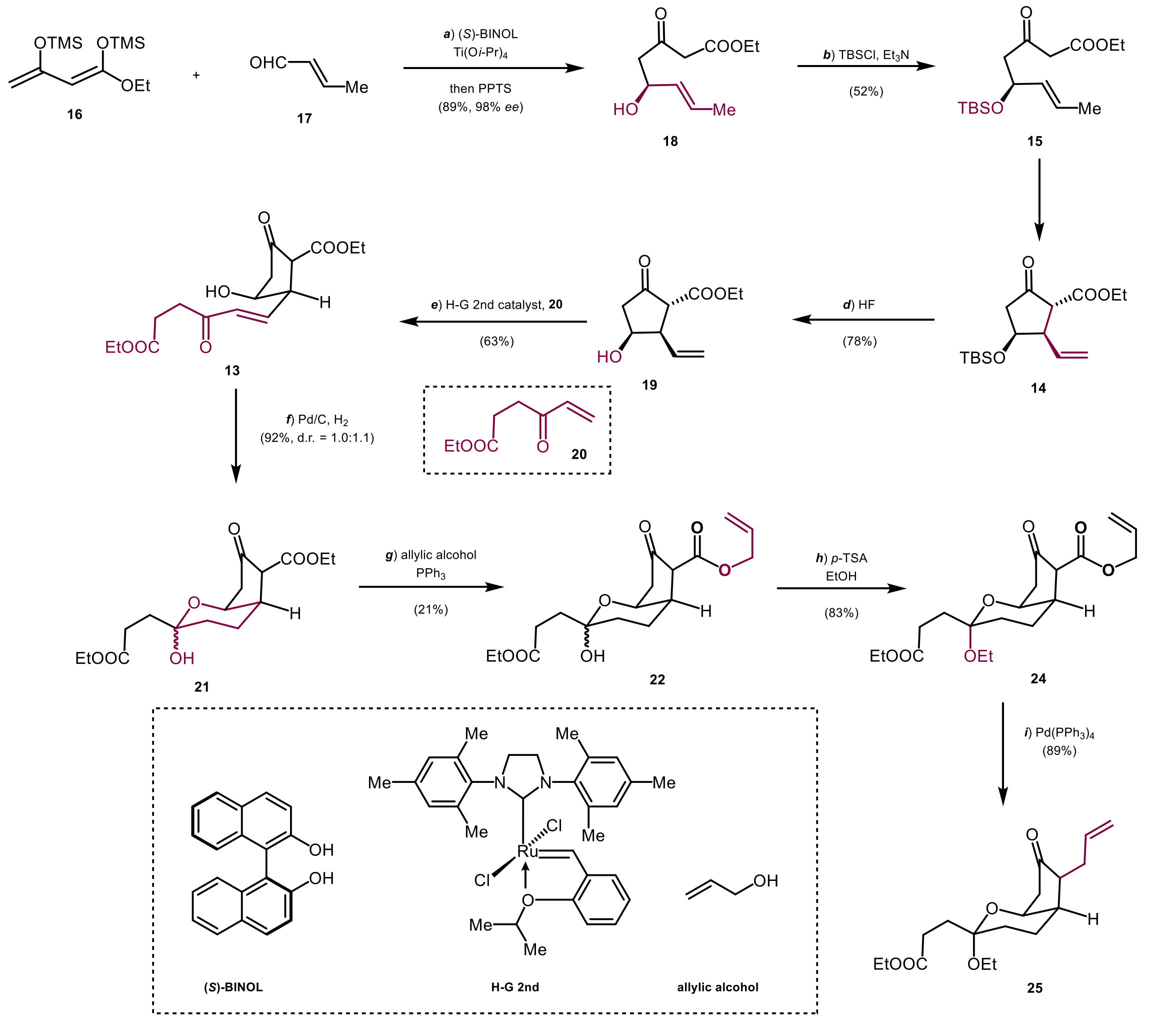

Scheme S1: Overview of the synthetic route toward compound **25**

Preparation of compound 18[1]:

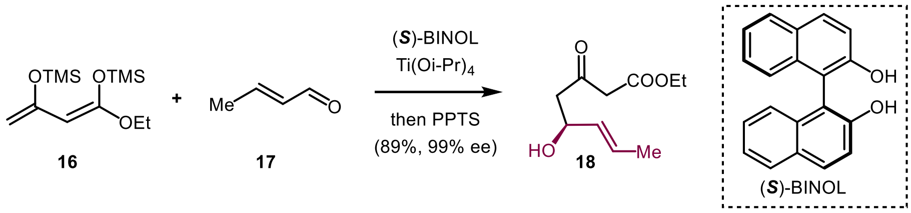

The mixture of $Ti(O\text{-}iPr)_4$ (0.29 mL, 0.98 mmol, 0.060 equiv) and (*S*)-BINOL (280 mg, 0.98 mmol, 0.060 equiv) in THF (10 mL) was stirred at room temperature for 20 min. The resultant Ti–BINOL complex was added to a solution of crotonaldehyde (**17**, 1.14g, 1.30 mL, 16.30 mmol, 1.00 equiv) and LiCl (165 mg, 3.91 mmol, 0.24 equiv) in THF (140 mL) at room temperature. After stirring at room temperature for 30 min, silyloxydiene **16** (13.41 g, 48.90 mmol, 3.00 equiv) was added to the mixture. The resultant mixture was stirred at room temperature for 14 h. After cooling to 0 °C, PPTS (819 mg, 3.26 mmol, 0.20 equiv) in MeOH (30 mL) was added and the mixture was stirred for 2 h. The reaction was quenched with $NaHCO_3$ (40 mL) and extracted with ethyl acetate (80 mL) for 3 times. The organic phase was dried over Na_2SO_4 and concentrated under vacuum. The resultant mixture was directly purified by flash chromatography on a silica column (hexane/EtOAc 4:1) to give product **18** (2.91g, 89%) as a yellow oil.

Compound 18: R$_f$ = 0.3 (silica gel, hexane/EtOAc 4:1). **$[\alpha]_D^{25}$** = -21.19 (c = 0.31, $CHCl_3$). **^{1}H NMR** (500 MHz, $CDCl_3$) δ 5.75 – 5.66 (m, 1H), 5.51 – 5.43 (m, 1H), 4.55 – 4.49 (m, 1H), 4.22 – 4.14 (m, 2H), 3.46 (s, 2H), 2.77 – 2.71 (m, 2H), 1.69 – 1.66 (m, 3H), 1.28 – 1.23 (m, 3H). **^{13}C NMR** (125 MHz, $CDCl_3$) δ 203.1, 167.1, 131.8, 127.6, 68.6, 61.6, 50.1, 49.8, 17.7, 14.2. **IR** (foam, KBr) 3471, 2983, 2942, 1737, 1716, 1318, 1029 cm^{-1}. **HRMS** (ESI): Calculated for $C_{10}H_{16}O_4Na^+$ $[M+Na]^+$: 223.0941, found: 223.0940. **HPLC analysis**: The ee was determined by HPLC on a CHIRALPAK AD-H column (*n*-hexane / EtOH = 93/7, flow rate = 1.0 mL/min, T = 25 °C, UV detection at λ = 254 nm); retention times for compound **18**: t_{R1} = 36.49 min (major), t_{R2} = 33.14 min (minor).

HPLC spectra of compound 18:

The ee was determined by HPLC on a CHIRALPAK AD-H column (*n*-hexane/EtOH 93/7, flow rate = 1.0 mL/min, *T* = 25 °C, UV detection at λ = 254 nm)

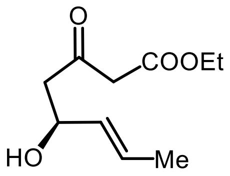

18
(98% ee)

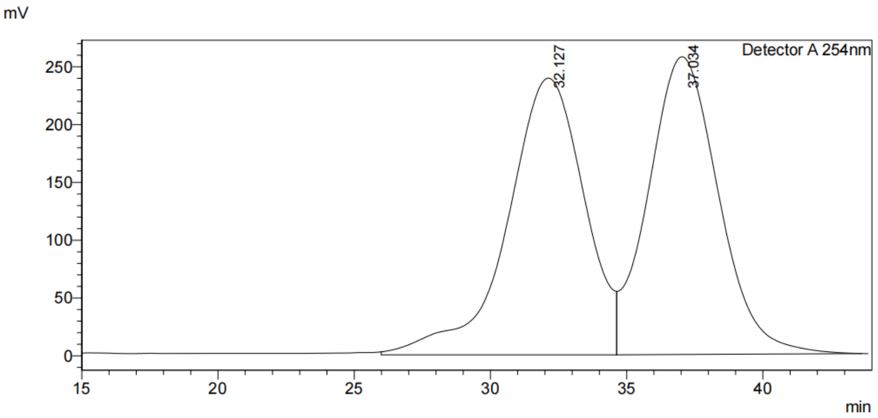

<Peak Table>

Detector A 254nm

Peak#	Ret. Time	Area	Height	Conc.	Unit	Mark	Name
1	32.127	46971079	239361	50.394		M	
2	37.034	46236144	257465	49.606		M	
Total		93207223	496826				

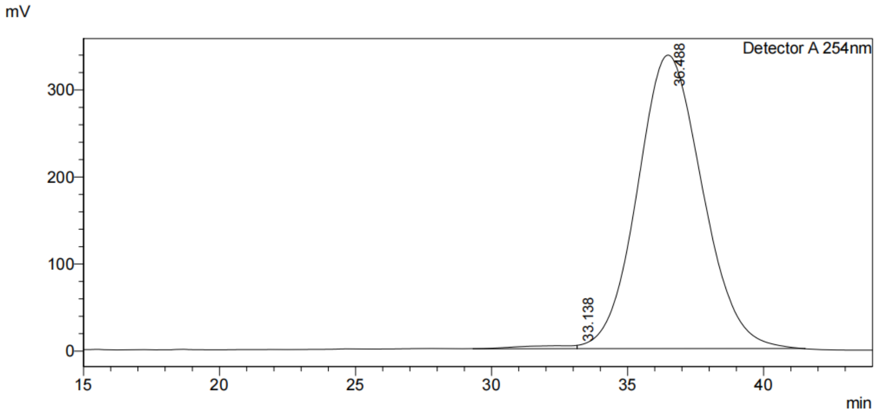

<Peak Table>

Detector A 254nm

Peak#	Ret. Time	Area	Height	Conc.	Unit	Mark	Name
1	33.138	504420	3785	0.890		M	
2	36.488	56156503	337235	99.110		M	
Total		56660923	341021				

Preparation of compound 15:

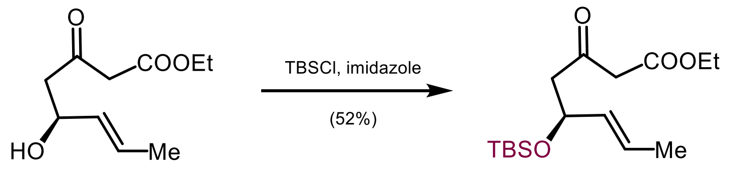

18 **15**

To a solution of compound **18** (2.00 g, 10.00 mmol, 1.00 equiv) in DCM (50 mL) was added imidazole (2.04 g, 30.00 mmol, 3.00 equiv) and TBSCl (2.26 g, 15.00 mmol, 1.50 equiv) at room temperature and the resultant mixture was stirred at room temperature for 10 h. The reaction was quenched with NH_4Cl (20 mL) and extracted with dichloromethane (20 mL) for 3 times. The organic phase was dried over Na_2SO_4 and concentrated under vacuum. The resultant mixture was directly purified by flash chromatography on a silica column (hexane/EtOAc 15:1) to give product **15** (1.63 g, 52%) as a yellow oil.

Compound 15: **R_f** = 0.50 (silica gel, hexane/EtOAc 15:1). **$[\alpha]_D^{25}$ =** -25.43 (c = 1.20, $CHCl_3$). **^{1}H NMR** (500 MHz, $CDCl_3$) δ 5.66 – 5.56 (m, 1H), 5.48 – 5.34 (m, 1H), 4.57 – 4.50 (m, 1H), 4.24 – 4.11 (m, 2H), 3.46 (s, 2H), 2.74 (dd, *J* = 14.8, 7.8 Hz, 1H), 2.54 (dd, *J* = 14.8, 4.7 Hz, 1H), 1.65 (dd, *J* = 6.5, 1.8 Hz, 3H), 1.27 – 1.24 (m, 3H), 0.84 (s, 9H), 0.01 (d, *J* = 7.4 Hz, 6H). **^{13}C NMR** (125 MHz, $CDCl_3$) δ 201.6, 167.2, 133.3, 126.4, 70.6, 61.4, 51.4, 51.0, 26.0 (3C), 18.2, 17.6, 14.2, -4.1, -4.9. **IR** (foam, KBr) 2955, 2929, 1749, 1721, 1652, 1472, 1252, 1070 cm^{-1}. **HRMS** (ESI): Calculated for $C_{16}H_{30}NaO_4SiNa^+$ $[M+Na]^+$: 337.1806, found: 337.1805.

Table S1. Optimization of reaction conditions[a]

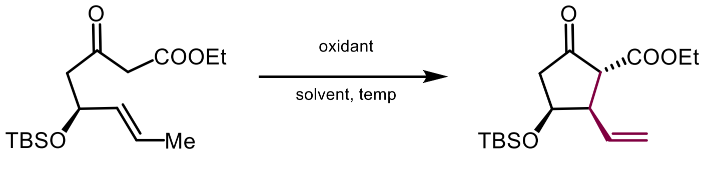

entry	oxidant	solvent	temp. (°C)	yield (%)[b]	d.r.[c]
1	$Mn(OAC)_3·2H_2O$ (2.2 eq), $Cu(OAC)_2·H_2O$ (1.1 eq)	MeCN	50	9	>20:1
2	$Mn(OAC)_3·2H_2O$ (2.2 eq), $Cu(OAC)_2·H_2O$ (1.1 eq)	EtOH	50	0	-
3	$Mn(OAC)_3·2H_2O$ (2.2 eq), $Cu(OAC)_2·H_2O$ (1.1 eq)	AcOH	50	12	>20:1
**4**	**$Mn(OAC)_3·2H_2O$ (2.2 eq), $Cu(OAC)_2·H_2O$ (1.1 eq)**	**HFIP**	**50**	**63**	**>20:1**
5	$Mn(OAC)_3·2H_2O$ (2.2 eq), $Cu(OAC)_2·H_2O$ (1.1 eq)	HFIP	70	trace	-
6	$Fe(ClO_4)_3·9H_2O$ (2.2 eq)	HFIP	50	0	-
7	CAN (2.2 eq)	HFIP	50	0	-

[a]Reaction conditions: All reactions were carried out with **15** (0.2 mmol), oxidant (220 mol %), and solvent (4 mL) unless otherwise stated. The reactions were conducted at 50 °C for 36 h with stirring under argon atmosphere. [b]Isolated yield. [c]Diastereomeric ratios (dr) was determined by ^{1}H NMR analysis.

Preparation of compound 14:

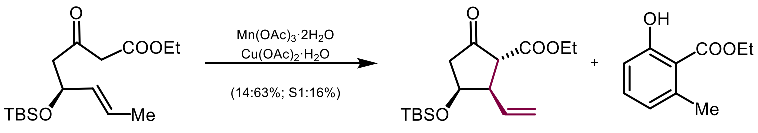

To a solution of compound **15** (1.57 g, 5.00 mmol, 1.00 equiv) in HFIP (100 mL) was added $Mn(OAc)_3·2H_2O$ (2.95 g, 11.00 mmol, 2.20 equiv) and $Cu(OAc)_2·H_2O$ (1.10 g, 5.50 mmol, 1.10 equiv) at room temperature, and the resultant mixture was stirred at 50 °C for 36 h. The reaction was quenched with H_2O (40 mL) and extracted with dichloromethane (40 mL) for 3 times. The organic phase was dried over Na_2SO_4 and concentrated under vacuum. The resultant mixture was directly purified by flash chromatography on a silica column (hexane/EtOAc 15:1) to give product **14** (0.98 g, 63%) as a yellow oil and product **S1** (144 mg, 16%) as a colorless oil.

Compound 14: $\mathbf{R_f}$ = 0.45 (silica gel, hexane/EtOAc 15:1). $\mathbf{[\alpha]_D^{25}}$ = +19.27 (c = 0.10, MeOH). $\mathbf{^1H}$ **NMR** (500 MHz, $CDCl_3$) δ 5.99 – 5.86 (m, 1H), 5.22 – 5.14 (m, 2H), 4.47 – 4.42 (m, 1H), 4.23 – 4.16 (m, 2H), 3.40 (d, J = 12.0 Hz, 1H), 3.23 – 3.14 (m, 1H), 2.53 (dd, J = 18.1, 4.2 Hz, 1H), 2.38 (d, J = 18.1 Hz, 1H), 1.29 – 1.25 (m, 3H), 0.86 – 0.85 (m, 9H), 0.04 (d, J = 7.0 Hz, 6H). $\mathbf{^{13}C}$ **NMR** (125 MHz, $CDCl_3$) δ 209.7, 169.1, 135.5, 117.9, 71.3, 61.5, 57.0, 51.7, 49.0, 25.8(3C), 18.1, 14.3, -4.7, -4.8. **IR** (foam, KBr) 2958, 2932, 1761, 1729, 1472, 1369, 1259, 1068 cm^{-1}. **HRMS** (ESI): Calculated for $C_{16}H_{28}O_4SiNa^+$ $[M+Na]^+$: 335.1649, found: 335.1648.

Compound S1: $\mathbf{R_f}$ = 0.85 (silica gel, hexane/EtOAc 15:1). $\mathbf{[\alpha]_D^{25}}$ = -3.30 (c = 0.20, $CHCl_3$). $\mathbf{^1H}$ **NMR** (500 MHz, $CDCl_3$) δ 11.38 (s, 1H), 7.30 – 7.27 (m, 1H), 6.85 (d, J = 8.3 Hz, 1H), 6.72 (d, J = 7.4 Hz, 1H), 4.49 – 4.40 (m, 2H), 2.56 (s, 3H), 1.46 – 1.41 (m, 3H). $\mathbf{^{13}C}$ **NMR** (125 MHz, $CDCl_3$) δ 171.9, 163.0, 141.5, 134.2, 123.0, 115.7, 112.6, 61.7, 24.2, 14.3. **IR** (foam, KBr) 3473, 2957, 2929, 1658, 1372, 1253, 1214, 1110 cm^{-1}. **HRMS** (ESI): Calculated for $C_{10}H_{12}O_3Na^+$ $[M+Na]^+$: 203.0679, found: 203.0680.

Preparation of compound 19:

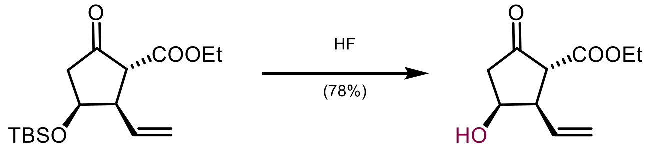

14 **19**

To a solution of compound **14** (1.40 g, 4.50 mmol, 1.00 equiv) in MeCN (22 mL) was added HF (3.0 mL, 40% in H_2O) at room temperature, and the resultant mixture was stirred at room temperature for 1 h. The reaction was quenched with $NaHCO_3$ (20 mL) and extracted with ethyl acetate (20 mL) for 3 times. The organic phase was dried over Na_2SO_4 and concentrated under vacuum. The resultant mixture was directly purified by flash chromatography on a silica column (hexane/EtOAc 4:1) to give product **19** (695 mg, 78%) as a yellow oil.

Compound 19: $\mathbf{R_f}$ = 0.40 (silica gel, hexane/EtOAc 4:1). $[\alpha]_D^{25}$ = -24.38 (c = 0.57, $CHCl_3$). **^{1}H NMR** (500 MHz, $CDCl_3$) δ 6.03 – 5.93 (m, 1H), 5.39 – 5.34 (m, 1H), 5.33 – 5.27 (m, 1H), 4.54 – 4.49 (m, 1H), 4.25 – 4.18 (m, 2H), 3.48 (d, *J* = 12.1 Hz, 1H), 3.38 – 3.30 (m, 1H), 2.58 – 2.53 (m, 2H), 1.30 – 1.26 (m, 3H). **^{13}C NMR** (125 MHz, $CDCl_3$) δ 209.2, 169.0, 133.9, 119.3, 69.7, 61.7, 55.6, 50.3, 47.6, 14.3. **IR** (foam, KBr) 3482, 2982, 2929, 1756, 1723, 1372, 1260, 1095 cm^{-1}. **HRMS** (ESI): Calculated for $C_{10}H_{14}O_4Na^+$ $[M+Na]^+$: 221.0784, found: 221.0785.

Preparation of compound 20[2]:

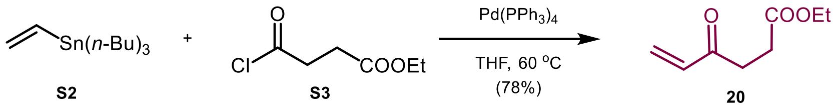

To a solution of ethyl succinyl chloride **S3** (8.67 mL, 60.80 mmol, 1.00 equiv) and tributylvinyltin **S2** (17.76 mL, 60.80 mmol, 1.00 equiv) in THF (60 mL) was added $Pd(PPh_3)_4$ (702 mg, 0.61 mmol, 0.01 equiv) at room temperature. The reaction was stirred at 60 °C for 2 days. The reaction was filtered and concentrated under vacuum. The resultant mixture was directly purified by flash chromatography on a silica column (hexane/EtOAc 8:1), to give product **20** (7.40 g, 78%) as a colorless oil.

Compound 20: $\mathbf{R_f}$ = 0.50 (silica gel, hexane/EtOAc 8:1). **^{1}H NMR** (500 MHz, $CDCl_3$) δ 6.41 – 6.31 (m, 1H), 6.29 – 6.21 (m, 1H), 5.88 – 5.82 (m, 1H), 4.13 – 4.10 (m, 2H), 2.94 – 2.87 (m, 2H), 2.63 – 2.58 (m, 2H), 1.25 – 1.21 (m, 3H). **^{13}C NMR** (125 MHz, $CDCl_3$) δ 198.7, 172.8, 136.3, 128.6, 60.7, 34.2, 28.0, 14.3 **IR** (foam, KBr) 2980, 2928, 1732, 1684, 1375, 1214, 1161 cm^{-1}. **HRMS** (ESI): Calculated for $C_8H_{12}O_3Na^+$ $[M+Na]^+$: 179.0679, found: 179.0680.

Preparation of compound 13:

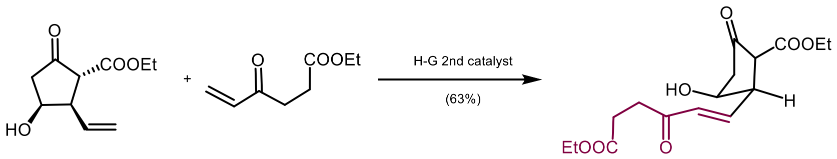

19 20 13

To a solution of compound **19** (693 mg, 3.50 mmol, 1.00 equiv) and ester **20** (2.19 g, 14.00 mmol, 4.00 equiv) in DCE (18 mL) was added Hoveyda–Grubbs II catalyst (110 mg, 0.18 mmol, 0.05 equiv) at room temperature, the resultant mixture was stirred at 60 °C for 20 h. The solvent was removed under vacuum and the resultant mixture was directly purified by flash chromatography on a silica column (hexane/EtOAc 1:1) to give product **13** (718 mg, 63%) as a brown oil.

Compound 13: R$_f$ = 0.40 (silica gel, hexane/EtOAc 1:1). **[α]$_D^{25}$** = -7.23 (c = 0.16, $CHCl_3$). **^{1}H NMR** (500 MHz, $CDCl_3$) δ 6.99 (dd, *J* = 16.0, 7.3 Hz, 1H), 6.29 (dd, *J* = 16.0, 1.2 Hz, 1H), 4.62 – 4.58 (m, 1H), 4.25 – 4.16 (m, 2H), 4.15 – 4.10 (m, 2H), 3.52 (d, *J* = 11.9 Hz, 1H), 3.45 – 3.38 (m, 1H), 2.93 – 2.88 (m, 2H), 2.64 – 2.61 (m, 3H), 2.54 (d, *J* = 18.5 Hz, 1H), 1.29 – 1.24 (m, 6H). **^{13}C NMR** (125 MHz, $CDCl_3$) δ 208.1, 198.1, 173.2, 168.4, 142.7, 132.1, 69.8, 62.0, 60.9, 56.0, 49.3, 47.9, 35.2, 28.1, 14.3, 14.3. **IR** (foam, KBr) 3499, 2926, 1760, 1678, 1373, 1208, 1164, 1021 cm^{-1}. **HRMS** (ESI): Calculated for $C_{16}H_{22}O_7Na^+$ $[M+Na]^+$: 349.1258, found: 349.1260.

Preparation of compound 21:

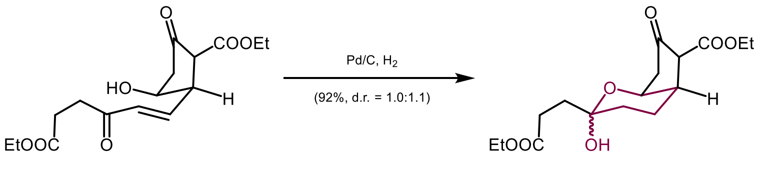

13 21

To a solution of compound **13** (718 mg, 2.20 mmol, 1.00 equiv) in EtOAc (55 mL) was added Pd/C (222 mg, 0.22 mmol, 0.10 equiv) at room temperature, and the resultant mixture was stirred at room temperature for 2 h. The solvent was filtered and concentrated under vacuum and the resultant mixture was directly purified by flash chromatography on a silica column (hexane/EtOAc 1:1) to give product **21** (664 mg, 92%) as a yellow oil.

Compound 21: **R_f** = 0.30 (silica gel, hexane/EtOAc 1:1). **$[\alpha]_D^{25}$** = -22.57 (c = 0.14, $CHCl_3$). **^{1}H NMR** (500 MHz, $CDCl_3$) δ 4.26 – 4.17 (m, 3H), 4.16 – 4.09 (m, 2H), 3.26 (dd, J = 53.8, 12.0 Hz, 1H), 2.83 – 2.71 (m, 1H), 2.70 – 2.49 (m, 3H), 2.48 (d, J = 2.7 Hz, 1H), 2.43 – 2.37 (m, 1H), 2.34 – 2.19 (m, 1H), 2.10 – 1.98 (m, 1H), 1.96 – 1.83 (m, 1H), 1.71 – 1.66 (m, 1H), 1.61 – 1.49 (m, 1H), 1.29 – 1.25 (m, 6H). **^{13}C NMR** (125 MHz, $CDCl_3$) δ 211.2, 210.5, 209.6, 175.6, 172.9, 169.6, 169.5, 95.5, 68.1, 67.4, 61.7, 61.7, 61.2, 61.0, 57.7, 54.1, 47.7, 46.8, 41.2, 38.7, 37.0, 36.8, 28.3, 28.3, 28.0, 21.0, 19.1, 14.4, 14.3, 14.3. **IR** (foam, KBr) 3478, 2920,1716, 1645, 1375, 1260, 1185, 1029 cm^{-1}. **HRMS** (ESI): Calculated for $C_{16}H_{24}O_7Na^+$ $[M+Na]^+$: 351.1414, found: 351.1415.

Preparation of compound 22:

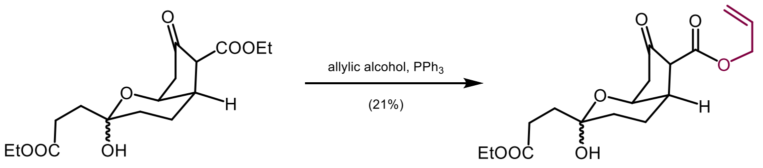

21 22

To a solution of compound **21** (460 mg, 1.40 mmol, 1.00 equiv) in PhMe (20 mL) was added allylic alcohol (1.63 g, 28.00 mmol, 20.00 equiv) and PPh_3 (73 mg, 0.28 mmol, 0.20 equiv) at room temperature and the mixture was stirred at 128 °C for 2 h. The solvent was removed under vacuum and the resultant mixture was purified by flash chromatography on a silica column (hexane/EtOAc 1:1) to give product **22** (100 mg, 21%) as a yellow oil.

Compound 22: **R_f** = 0.55 (silica gel, hexane/EtOAc 1:1). **^{1}H NMR** (500 MHz, CD_3OD) δ 6.00 – 5.87 (m, 1H), 5.40 – 5.32 (m, 1H), 5.27 – 5.20 (m, 1H), 4.71 – 4.63 (m, 2H), 4.40 (d, J = 4.1 Hz, 1H), 4.14 – 4.05 (m, 2H), 2.78 – 2.74 (m, 1H), 2.63 (dd, J = 16.4, 1.7 Hz, 2H), 2.59 – 2.48 (m, 3H), 2.44 – 2.38 (m, 1H), 2.36 (d, J = 18.4 Hz, 1H), 2.25 (s, 1H), 1.95 – 1.79 (m, 2H), 1.68 (d, J = 11.4 Hz, 1H), 1.26 – 1.21 (m, 3H). **^{13}C NMR** (125 MHz, CD_3OD) δ 212.1, 211.0, 175.6, 174.6, 171.1, 170.7, 133.3,

133.3, 118.6, 118.5, 97.0, 69.2, 69.0, 66.9, 66.8, 61.7, 61.6, 47.5, 47.4, 40.8, 40.0, 38.5, 37.8, 29.7, 28.9, 27.2, 23.8, 19.8, 14.5, 14.5. **IR** (foam, KBr) 3481, 2961, 2932, 1715, 1446, 1373, 1260, 1029, 939, 797, 735 cm^{-1}. **HRMS** (ESI): Calculated for $C_{17}H_{24}O_7Na^+$ $[M+Na]^+$: 363.1414, found: 363.1413.

Preparation of compound 24:

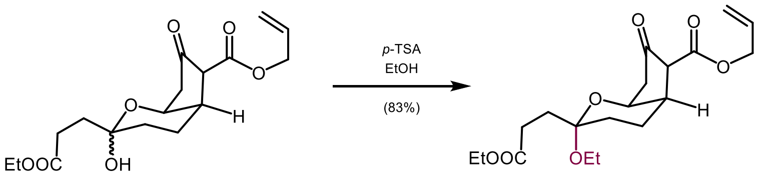

22 **24**

To a solution of compound **22** (100 mg, 0.29 mmol, 1.00 equiv) in EtOH (3 mL) was added *p*-TSA (5 mg, 0.03 mmol, 0.10 equiv) and 4 Å molecular sieves (50 mg) at room temperature and the mixture was stirred at room temperature for 5 h. The mixture was filtered and concentrated under vacuum. The resultant mixture was directly purified by flash chromatography on a silica column (hexane/EtOAc 1:1) to give product **24** (89 mg, 83%) as a colorless oil.

Compound 24: $\mathbf{R_f}$ = 0.55 (silica gel, hexane/EtOAc 1:1). **^{1}H NMR** (500 MHz, $CDCl_3$) δ 5.98 – 5.89 (m, 1H), 5.38 – 5.31 (m, 1H), 5.25 – 5.19 (m, 1H), 4.68 – 4.59 (m, 2H), 4.34 (dd, J = 4.8, 3.4 Hz, 1H), 4.14 – 4.04 (m, 2H), 3.52 – 3.43 (m, 2H), 2.68 – 2.55 (m, 2H), 2.40 – 2.27 (m, 3H), 2.28 – 2.17 (m, 1H), 2.00 – 1.87 (m, 2H), 1.69 – 1.55 (m, 3H), 1.29 (s, 1H), 1.25 – 1.23 (m, 3H), 1.22 – 1.18 (m, 3H). **^{13}C NMR** (125 MHz, $CDCl_3$) δ 211.8, 175.1, 170.6, 133.3, 118.5, 100.2, 69.8 (2C), 66.8, 61.6, 56.5, 47.3, 39.9, 32.8, 29.8, 26.6, 19.6, 15.6, 14.5. **IR** (foam, KBr) 3457, 2978, 2933, 1759, 1728, 1648, 1445, 1376, 1283, 1238, 1180, 1130, 1056, 1025, 990, 857, 801, 746 cm^{-1}. **HRMS** (ESI): Calculated for $C_{19}H_{28}O_7Na^+$ $[M+Na]^+$: 391.1727, found: 391.1726.

Preparation of compound 25[1]:

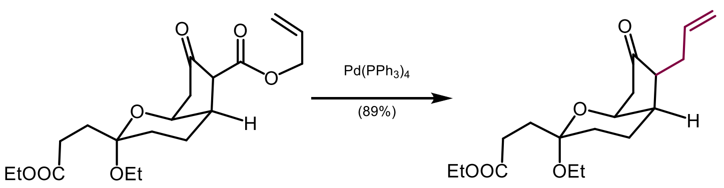

To a solution of compound **24** (88 mg, 0.24 mmol, 1.00 equiv) in PhMe (5 mL) was added $Pd(PPh_3)_4$ (14 mg, 0.012 mmol, 0.05 equiv) at room temperature and the resultant mixture was stirred at 90 °C for 12 h. The mixture was concentrated under vacuum. The resultant mixture was directly purified by flash chromatography on a silica column (hexane/EtOAc 4:1) to give product **25** (69 mg, 89%) as a colorless oil.

Compound 25: $\mathbf{R_f}$ = 0.56 (silica gel, hexane/EtOAc 4:1). $[\alpha]_D^{25}$ = -84.177 (c = 0.30, $CHCl_3$). **^{1}H NMR** (500 MHz, $(CD_3)_2CO$) δ 5.85 – 5.69 (m, 1H), 5.09 – 4.94 (m, 2H), 4.27 (dd, J = 4.9, 3.4 Hz, 1H), 4.11 – 4.00 (m, 2H), 3.53 – 3.39 (m, 2H), 2.41 – 2.26 (m, 6H), 2.20 – 2.08 (m, 2H), 2.03 – 1.98 (m, 1H), 1.95 – 1.80 (m, 2H), 1.76 – 1.69 (m, 1H), 1.62 (d, J = 8.8 Hz, 1H), 1.58 – 1.49 (m, 1H), 1.25 – 1.13 (m, 6H). **^{13}C NMR** (125 MHz, $(CD_3)_2CO$) δ 218.0, 173.6, 137.0, 117.1, 99.5, 69.6, 60.7, 55.8, 46.5, 46.5, 39.6, 33.6, 32.6, 26.6, 21.1, 19.2, 15.7, 14.6. **IR** (foam, KBr) 1295, 1742, 1449, 1327, 1298, 1183, 1025 cm^{-1}. **HRMS** (ESI): Calculated for $C_{18}H_{28}O_5Na^+$ $[M+Na]^+$: 347.1829, found: 347.1828.

3. Second generation asymmetric total synthesis of tricyclic-PGDM methyl ester

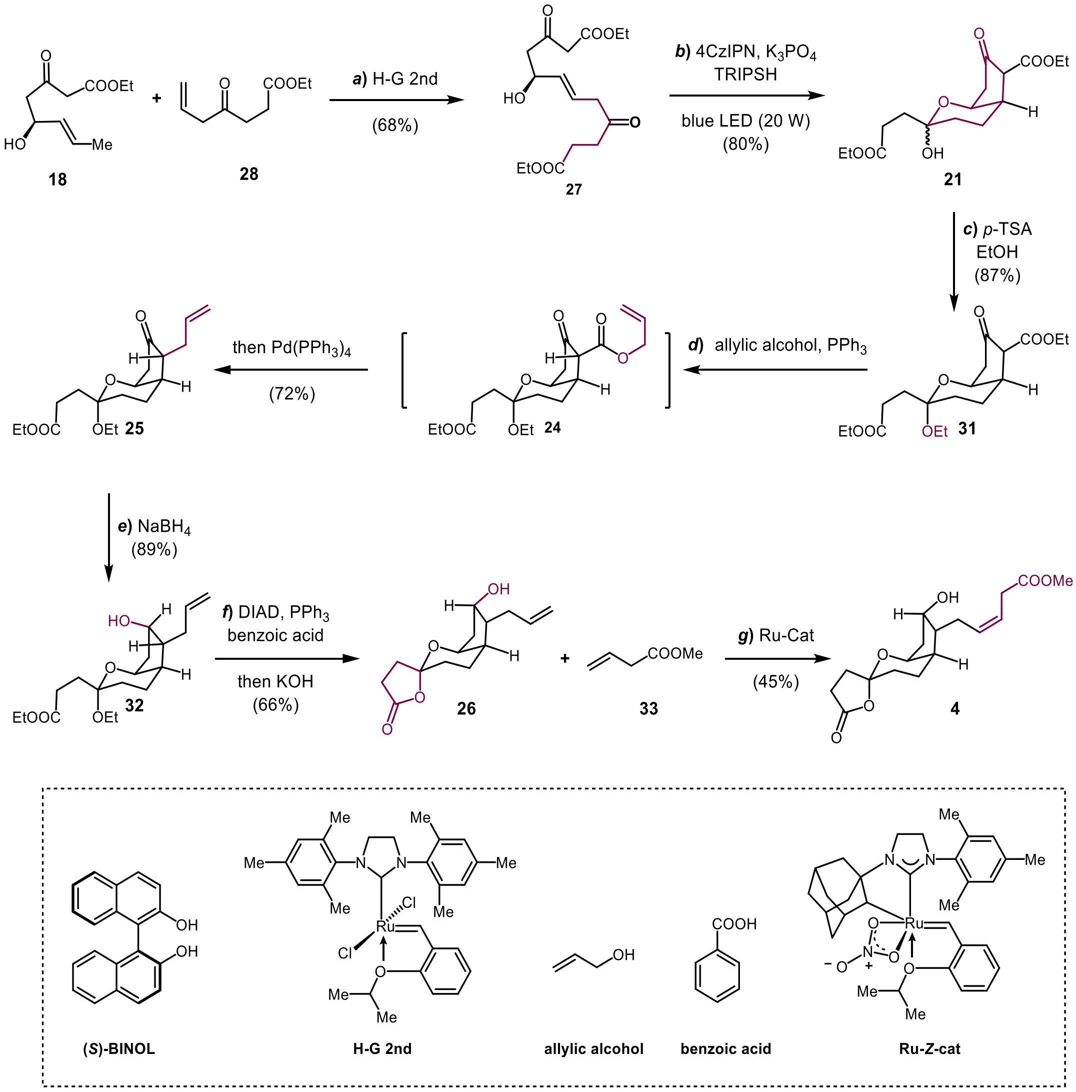

Scheme S2: Overview of the synthetic route for the asymmetric synthesis of tricyclic-PGDM methyl ester **4**

Preparation of compound 28[2]:

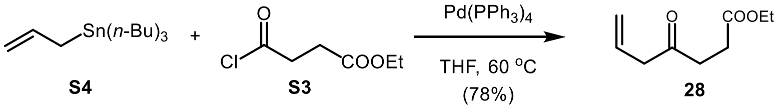

To a solution of ethyl succinyl chloride **S3** (8.60 mL, 60.80 mmol, 1.00 equiv) and allyltributyltin (**S4**, 18.80 mL, 60.80 mmol, 1.00 equiv) in THF (60 mL) was added $Pd(PPh_3)_4$ (702 mg, 0.61 mmol, 0.010 equiv) at room temperature and the reaction mixture was stirred at 60 °C for 2 days. The reaction was filtered and concentrated under vacuum. The residue was purified by flash chromatography (hexane/EtOAc 8:1) to give product **28** (8.07g, 78%) as a colorless oil.

Compound 28: $\mathbf{R_f}$ = 0.53 (silica gel, hexane/EtOAc 8:1). **^{1}H NMR** (500 MHz, $CDCl_3$) δ 5.97 – 5.87 (m, 1H), 5.22 – 5.12 (m, 2H), 4.17 – 4.07 (m, 2H), 3.22 (dd, J = 6.8, 1.6 Hz, 2H), 2.80 – 2.72 (m, 2H), 2.60 – 2.53 (m, 2H), 1.26 – 1.22 (m, 3H). **^{13}C NMR** (125 MHz, $CDCl_3$) δ 206.9, 172.9, 130.4, 119.2, 60.8, 47.8, 36.8, 28.1, 14.3. **IR** (foam, KBr) 2983, 2934, 1737, 1640, 1375, 1349, 1199, 1163 cm^{-1}. **HRMS** (ESI): Calculated for $C_9H_{15}O_3^+$ $[M+H]^+$: 171.1016, found: 171.1015.

Preparation of compound 27[1]:

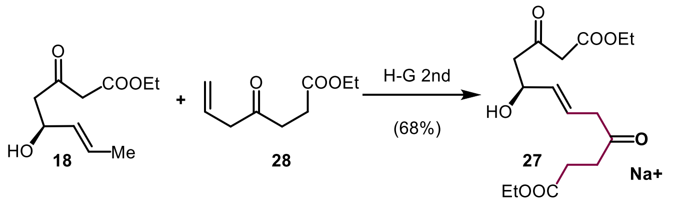

To a solution of compound **18** (2.00 g, 10.00 mmol, 1.00 equiv) and ester **28** (6.80 g, 40.00 mmol, 4.00 equiv) in DCE (30 mL) was added Hoveyda–Grubbs II catalyst (313 mg, 0.50 mmol, 0.050 equiv) at room temperature and the resultant mixture was stirred at 60 °C for 20 h. The solvent was removed under vacuum and the residue was purified by flash chromatography (hexane/EtOAc 1:1) to give product **27** (2.20 g, 68%, E/Z > 20:1) as a brown oil.

Compound 27: **R_f** = 0.47 (silica gel, hexane/EtOAc 1:1). **$[\alpha]_D^{25}$** = -4.53 (c = 0.50, $CHCl_3$). **^{1}H NMR** (500 MHz, $CDCl_3$) δ 5.91 – 5.79 (m, 1H), 5.68 – 5.56 (m, 1H), 4.61 (d, J = 6.1 Hz, 1H), 4.24 – 4.08 (m, 4H), 3.48 (s, 2H), 3.21 (d, *J* = 7.2 Hz, 2H), 2.84 – 2.70 (m, 4H), 2.62 – 2.52 (m, 2H), 1.29 – 1.22 (m, 6H). **^{13}C NMR** (125 MHz, $CDCl_3$) δ 206.7, 202.9, 172.8, 167.0, 135.1, 123.8, 68.1, 61.7, 60.8, 50.1, 49.4, 46.0, 36.8, 28.0, 14.3, 14.2. **IR** (foam, KBr) 3499, 2983, 2932, 1715, 1369, 1318, 1190, 1032 cm^{-1}. **HRMS** (ESI): Calculated for $C_{16}H_{24}O_7Na^+$ $[M+Na]^+$: 351.1414, found: 351.1415.

Preparation of compound 21[1]:

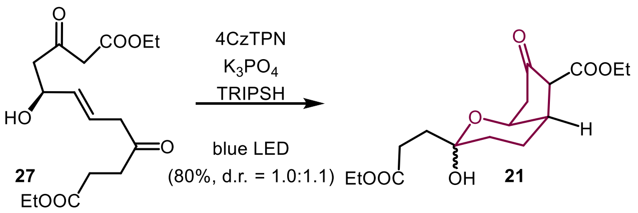

To a solution of compound **27** (800 mg, 2.44 mmol, 1.00 equiv) in THF (244 mL) was added K_3PO_4 (414 mg, 1.95 mmol, 0.80 equiv), TRIPSH (57 mg, 0.24 mmol, 0.10 equiv) and 4CzTPN (23 mg, 0.029 mmol, 0.02 equiv) at 25 °C. The mixture was irradiated with 20 W blue LED light (425 nm) at the same temperature for 30 min under argon. The solvent was removed under vacuum and the residue was purified by flash chromatography (hexane/EtOAc 1:1) to give product **21** (640 mg, 80%) as a yellow oil.

Preparation of compound 31[1]:

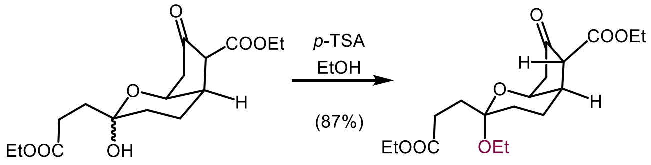

21 **31**

To a solution of compound **21** (650 mg, 1.98 mmol, 1.00 equiv) in EtOH (20 mL) was added *p*-TSA (34 mg, 0.20 mmol, 0.10 equiv) and 4 Å molecular sieves (50 mg) at

room temperature and the mixture was stirred at room temperature for 5 h. The mixture was filtered and concentrated under vacuum and the residue was purified by flash chromatography (hexane/EtOAc 4:1) to give product **31** (613 mg, 87%) as a yellow oil.

Compound 31: R_f = 0.45 (silica gel, hexane/EtOAc 4:1). **$[\alpha]_D^{25}$ = -53.6** (c = 0.94, $CHCl_3$). **^{1}H NMR** (500 MHz, $CDCl_3$) δ 4.29 – 4.22 (m, 1H), 4.22 – 4.11 (m, 2H), 4.11 – 4.02 (m, 2H), 3.48 – 3.36 (m, 2H), 3.29 (d, *J* = 12.0 Hz, 1H), 2.67 – 2.58 (m, 1H), 2.47 (dd, *J* = 18.5, 4.8 Hz, 1H), 2.36 (d, *J* = 18.5 Hz, 1H), 2.31 – 2.15 (m, 3H), 1.97 – 1.79 (m, 2H), 1.67 – 1.47 (m, 3H), 1.28 – 1.12 (m, 9H). **^{13}C NMR** (125 MHz, $CDCl_3$) δ 210.0, 173.3, 169.3, 98.8, 68.3, 61.6, 60.6, 55.5, 54.0, 46.6, 38.4, 31.8, 28.9, 25.8, 18.6, 15.3, 14.3, 14.2. **IR** (foam, KBr) 2924, 2851, 1731, 1375, 1290, 1183, 1130, 1022 cm^{-1}. **HRMS** (ESI): Calculated for $C_{18}H_{28}O_7Na^+$ $[M+Na]^+$: 379.1727, found: 379.1728. **HPLC analysis**: The ee was determined by HPLC on a CHIRALPAK AD-H column (*n*-hexane / EtOH = 90/10, flow rate = 1.0 mL/min, T = 27 °C, UV detection at λ = 210 nm); retention times for compound 31: t_{R1} = 9.84 min (major), t_{R2} = 17.18 min (minor).

HPLC spectra of compound 31:

The ee was determined by HPLC on a CHIRALPAK AD-H column (n-hexane/EtOH 90:10, flow rate = 1.0 mL/min, T = 27 °C, UV detection at λ = 210 nm)

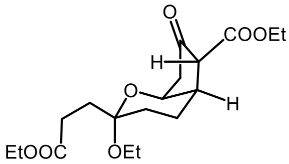

31
(97% ee)

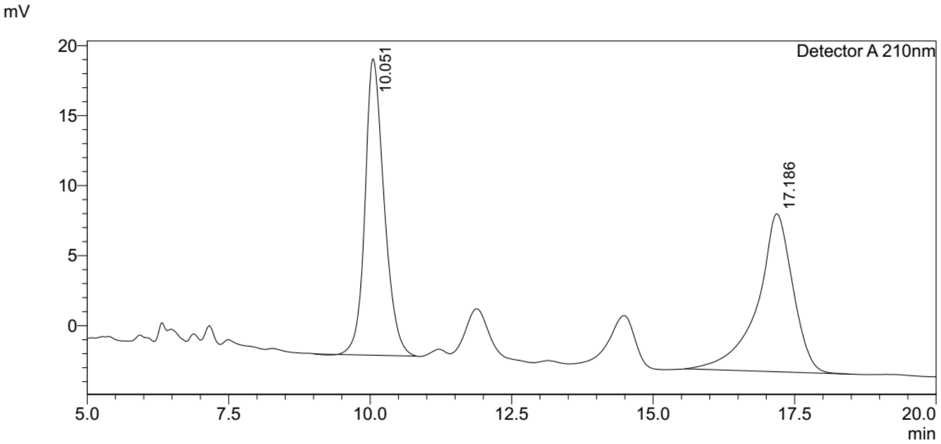

<Peak Table>

Detector A 210nm

Peak#	Ret. Time	Area	Height	Conc.	Unit	Mark	Name
1	10.051	499252	21178	50.422		M	
2	17.186	490893	11284	49.578		M	
Total		990145	32463				

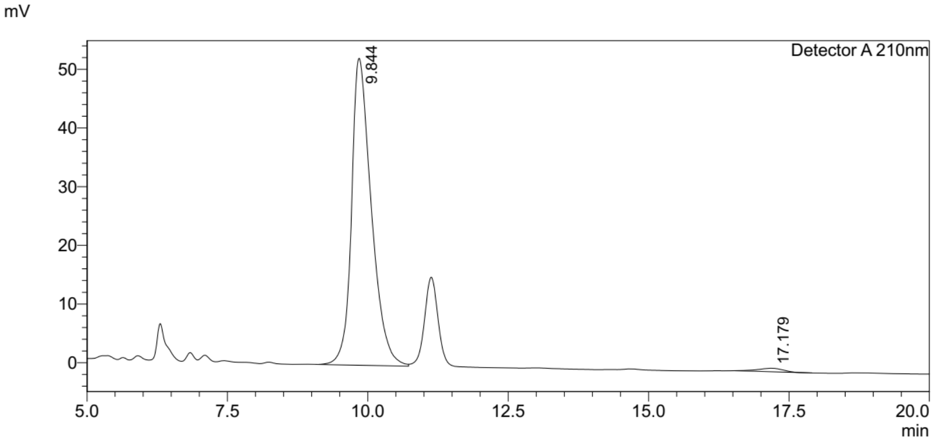

<Peak Table>

Detector A 210nm

Peak#	Ret. Time	Area	Height	Conc.	Unit	Mark	Name
1	9.844	1283848	52316	98.593			
2	17.179	18321	588	1.407		M	
Total		1302169	52905				

Preparation of compound 25[1]:

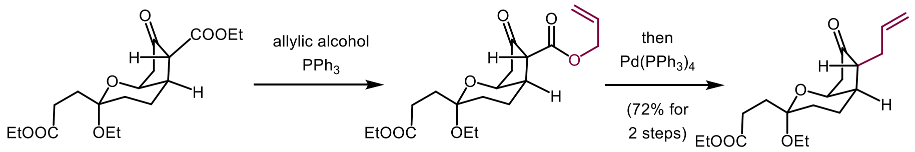

31 **24** **25**

To a solution of compound **31** (500 mg, 1.40 mmol, 1.00 equiv) in PhMe (20 mL) was added allylic alcohol (1.626 g, 28.00 mmol, 20.00 equiv) and PPh_3 (74 mg, 0.28 mmol, 0.20 equiv) at room temperature. The mixture was stirred at 128 °C for 2 h. After cooling the reaction to room temperature, $Pd(PPh_3)_4$ (80 mg, 0.070 mmol, 0.050 equiv) was added at room temperature and the mixture was stirred at room temperature for 12 h. The resultant mixture was purified by flash chromatography (hexane/EtOAc 4:1) to give product **25** (328 mg, 72%) as a colorless oil.

Preparation of compound 32[1]:

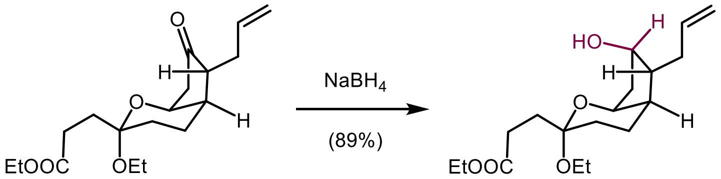

25 **32**

To a solution of compound **25** (300 mg, 0.93 mmol, 1.00 equiv) in EtOH (10 mL) was added $NaBH_4$ (11 mg, 0.28 mmol, 0.30 equiv) at 0 °C and the mixture was stirred at the same temperature for 5 h. Then solvent was removed under vacuum and the residue was purified by flash column chromatography (hexane/EtOAc 4:1) to give product **32** (268 mg, 89%) as a colorless oil.

Compound 32: $\mathbf{R_f}$ = 0.52 (silica gel, hexane/EtOAc 4:1). $\mathbf{[\alpha]_D^{25}}$ = -27.85 (c = 0.32, $CHCl_3$). **^{1}H NMR** (500 MHz, $(CD_3)_2CO$) δ 5.97 – 5.85 (m, 1H), 5.11 – 4.91 (m, 2H), 4.14 – 4.03 (m, 2H), 4.03 – 3.94 (m, 1H), 3.85 – 3.76 (m, 1H), 3.46 – 3.38 (m, 2H), 2.87 (d, J = 16.5 Hz, 2H), 2.36 – 2.26 (m, 2H), 2.23 – 2.18 (m, 1H), 2.15 – 2.08 (m, 1H), 2.02 – 1.84 (m, 4H), 1.66 – 1.40 (m, 5H), 1.20 (d, J = 7.1 Hz, 3H), 1.17 – 1.11 (m, 3H). **^{13}C NMR** (125 MHz, $(CD_3)_2CO$) δ 173.7, 138.2, 116.0, 99.6, 77.3, 77.2,

73.5, 60.7, 48.7 (2C), 42.5, 41.8, 36.8, 32.6, 28.2, 19.5, 15.7, 14.5. **IR** (foam, KBr) 3456, 2975, 2932, 1732, 1640, 1445, 1373, 1180 cm^{-1}. **HRMS** (ESI): Calculated for $C_{18}H_{30}O_5Na^+$ $[M+Na]^+$: 347.1985, found: 347.1987.

Preparation of compound 26[1]:

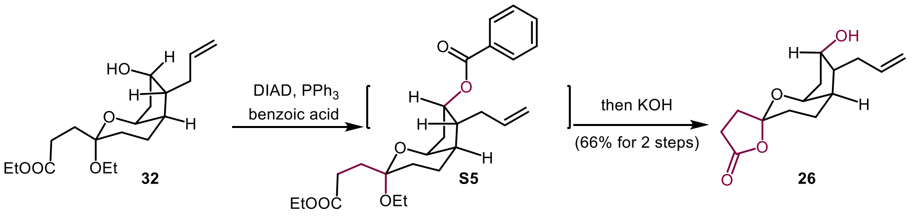

To a solution of compound **32** (300 mg, 0.92 mmol, 1.00 equiv) in THF (18 mL) was added PPh_3 (724 mg, 2.76 mmol, 3.00 equiv) and benzoic acid (124 mg, 1.01 mmol, 1.10 equiv) at room temperature. After cooling the mixture to −40 °C, DIAD (558 mg, 543 µL, 2.76 mmol, 3.00 equiv) was added and the mixture stirred at the same temperature for 40 min. After that, the mixture was allowed to warm to room temperature and stirred for 12 h. The solvent was removed under vacuum, and the crude **S5** was used in the next step without further purification.

To the above crude **S5** in MeOH (10 mL) was added KOH (5.00 M in H_2O, 2.00 mL) at room temperature and the mixture was degassed with Ar atmosphere for three times. The mixture was stirred at 45 °C for 4 h. The reaction was quenched with HCl (2.00 M in H_2O, 5.50 mL), and the mixture was extracted with diethyl ether for 3 times. The combined extracts were dried over Na_2SO_4, the solvent removed under vacuum and the residue was purified by flash column chromatography (hexane/EtOAc 1:1) to give product **26** (153 mg, 66%) as a colorless oil.

Compound S5: R_f = 0.43 (silica gel, hexane/EtOAc 1:1). **^{1}H NMR** (500 MHz, $(CD_3)_2CO$) δ 8.08 – 7.98 (m, 2H), 7.63 (d, J = 7.4 Hz, 1H), 7.59 – 7.48 (m, 2H), 5.87 (d, J = 6.8 Hz, 1H), 5.54 – 5.46 (m, 1H), 5.02 – 4.86 (m, 2H), 4.26 – 4.16 (m, 1H), 4.09 (q, *J* = 7.1 Hz, 2H), 3.51 – 3.37 (m, 2H), 2.89 – 2.84 (m, 2H), 2.33 – 2.28 (m, 4H), 2.19 – 2.10 (m, 2H), 2.00 – 1.85 (m, 3H), 1.68 – 1.54 (m, 2H), 1.49 – 1.42 (m,

1H), 1.22 (t, *J* = 7.1 Hz, 3H), 1.14 (t, *J* = 7.1 Hz, 3H).. **^{13}C NMR** (125 MHz, $(CD_3)_2CO$) δ 173.6, 166.2, 138.2, 133.8, 131.7, 130.2, 129.4, 116.0, 99.7, 77.1, 72.8, 60.7, 55.7, 43.4, 42.4, 41.2, 33.0, 32.5, 28.1, 19.1, 15.7, 14.6. **IR** (foam, KBr) 2976, 2930, 1716, 1641, 1452, 1275, 1027 cm^{-1}. **HRMS** (ESI): Calculated for $C_{25}H_{34}O_6Na^+$ $[M+Na]^+$:453.2248, found: 453.2247.

Compound 26: R$_f$ = 0.73 (silica gel, hexane/EtOAc 1:1). **$[\alpha]_D^{25}$** = -3.44 (c = 0.25, $CHCl_3$). **^{1}H NMR** (500 MHz, $CDCl_3$) δ 5.98 – 5.88 (m, 1H), 5.17 (dd, *J* = 16.9, 2.9 Hz, 1H), 5.07 (d, *J* = 10.1 Hz, 1H), 4.44 – 4.34 (m, 2H), 2.82 – 2.70 (m, 1H), 2.55 – 2.45 (m, 1H), 2.25 – 2.14 (m, 4H), 2.06 – 2.00 (m, 3H), 1.93 – 1.84 (m, 2H), 1.74 – 1.70 (m, 3H), 1.68 – 1.64 (m, 1H). **^{13}C NMR** (125 MHz, $CDCl_3$) δ 176.8, 137.9, 116.0, 108.2, 72.7, 42.9, 42.2, 39.9, 34.9, 31.9, 28.8, 28.5, 18.6. **IR** (foam, KBr) 3486, 2924, 2850, 1771, 1262, 1094, 1036, 904 cm^{-1}. **HRMS** (ESI): Calculated for $C_{14}H_{21}O_4^+$ $[M+H]^+$: 253.1434, found: 253.1437.

Preparation of compound 4[1]:

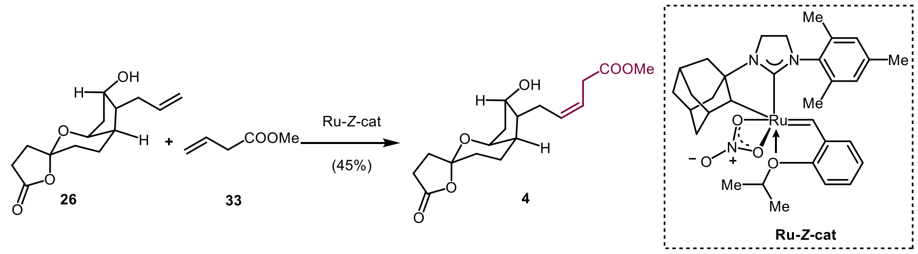

To a solution of compound **26** (100 mg, 0.40 mmol, 1.00 equiv) in DCE (4.0 mL) was added ester **33** (320 mg, 3.20 mmol, 8.00 equiv) and Ru-*Z*-catalyst (51 mg, 0.080 mmol, 0.20 equiv) at room temperature and the mixture was stirred at 40 °C for 12 h. The solvent was removed under vacuum to half. The resultant residue was purified by flash chromatography (hexane/EtOAc 1:2) to give product **4** (58 mg, 45%, *E/Z* = 1:16) as a brown oil.

Compound 4: $\mathbf{R_f}$ = 0.82 (silica gel, hexane/EtOAc 1:2). $[\alpha]_D^{25}$ = +48.00 (c = 0.05, $CHCl_3$). **^{1}H NMR** (500 MHz, $CDCl_3$) δ 5.73 – 5.64 (m, 1H), 5.61 – 5.52 (m, 1H), 4.45 – 4.38 (m, 1H), 4.38 – 4.26 (m, 1H), 3.70 (s, 3H), 3.35 – 3.25 (m, 1H), 3.03 (dd, *J* = 15.9, 6.2 Hz, 1H), 2.81 – 2.71 (m, 1H), 2.52 – 2.44 (m, 1H), 2.38 – 2.28 (m, 1H), 2.28 – 2.14 (m, 2H), 2.11 – 1.62 (m, 10H). **^{13}C NMR** (125 MHz, $CDCl_3$) δ 176.8, 173.3, 133.1, 121.8, 108.2, 75.1, 72.0, 52.4, 43.8, 42.3, 39.4, 35.04, 33.1, 28.8, 28.5, 25.1, 18.5. **IR** (foam, KBr) 3463, 2924, 2856, 1770, 1734, 1259, 1242, 1032 cm^{-1}. **HRMS** (ESI): Calculated for $C_{17}H_{24}O_6Na^+$ $[M+Na]^+$: 347.1465, found: 347.1469.

4. NMR spectra for the synthesized compounds

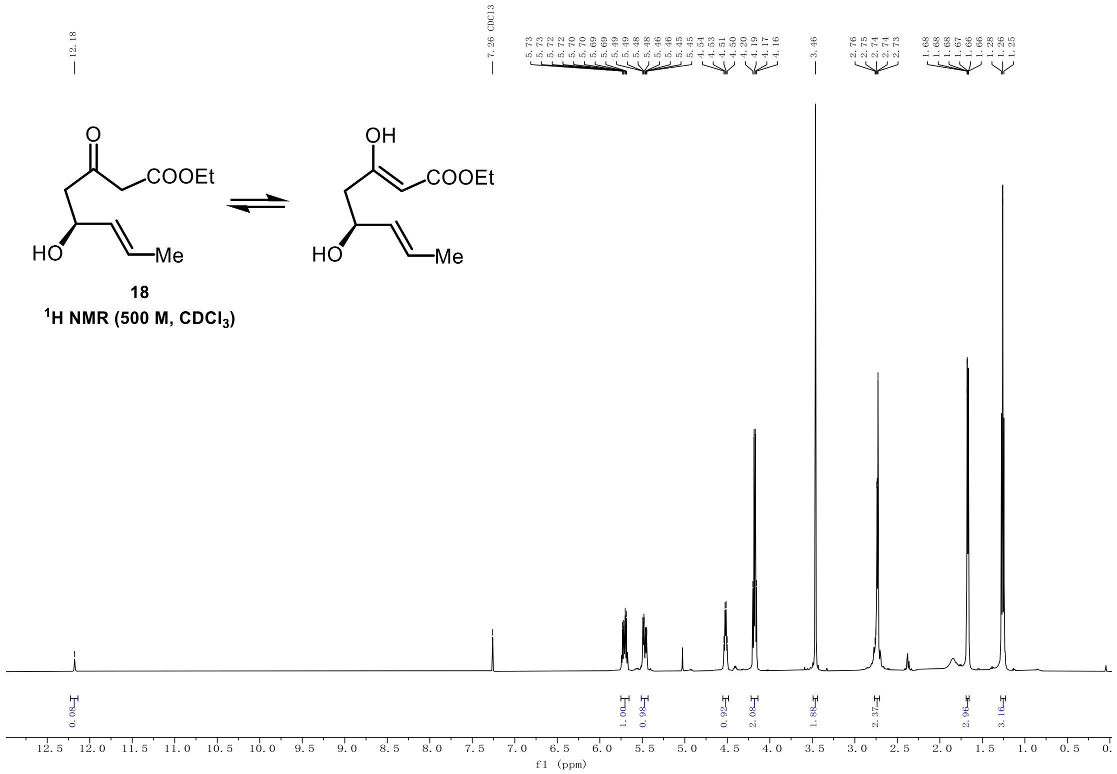

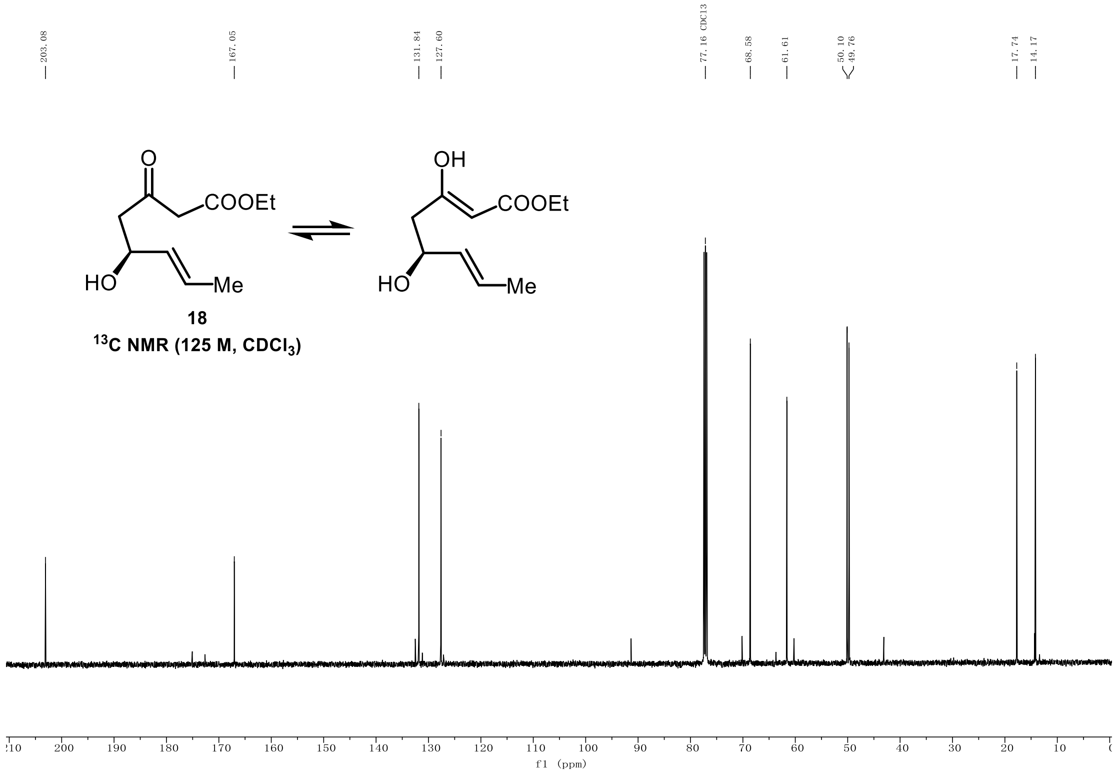

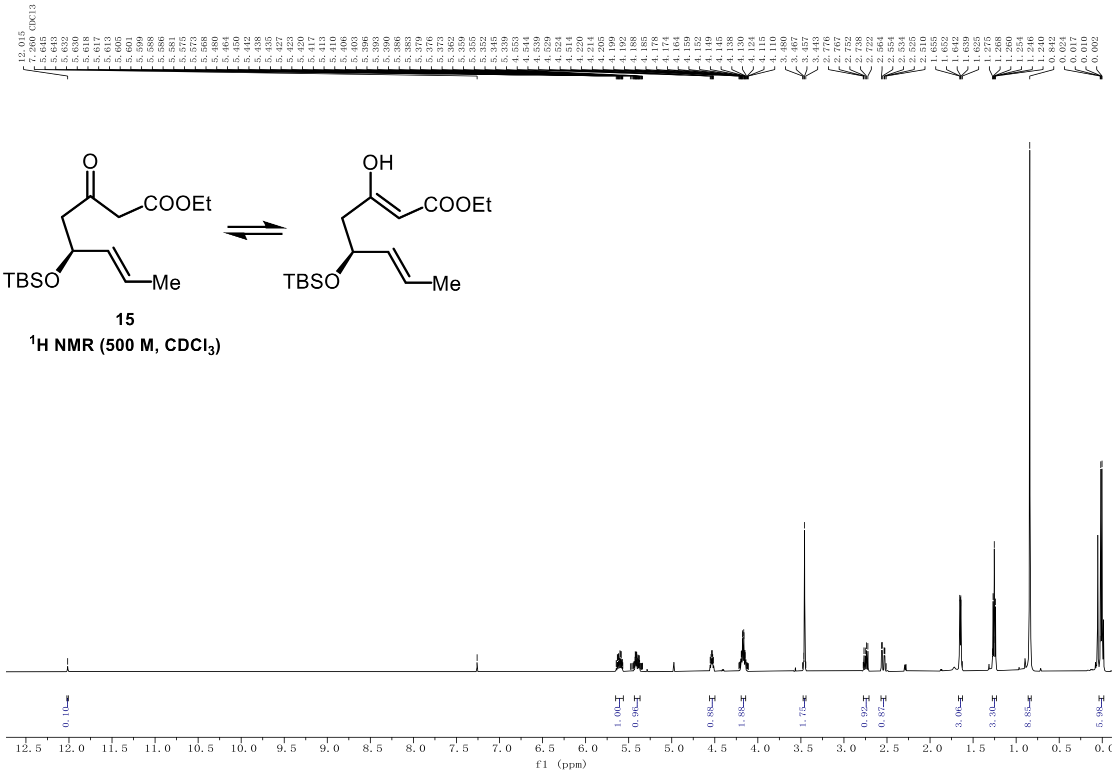

15

^{1}H NMR (500 M, CDCl$_3$)

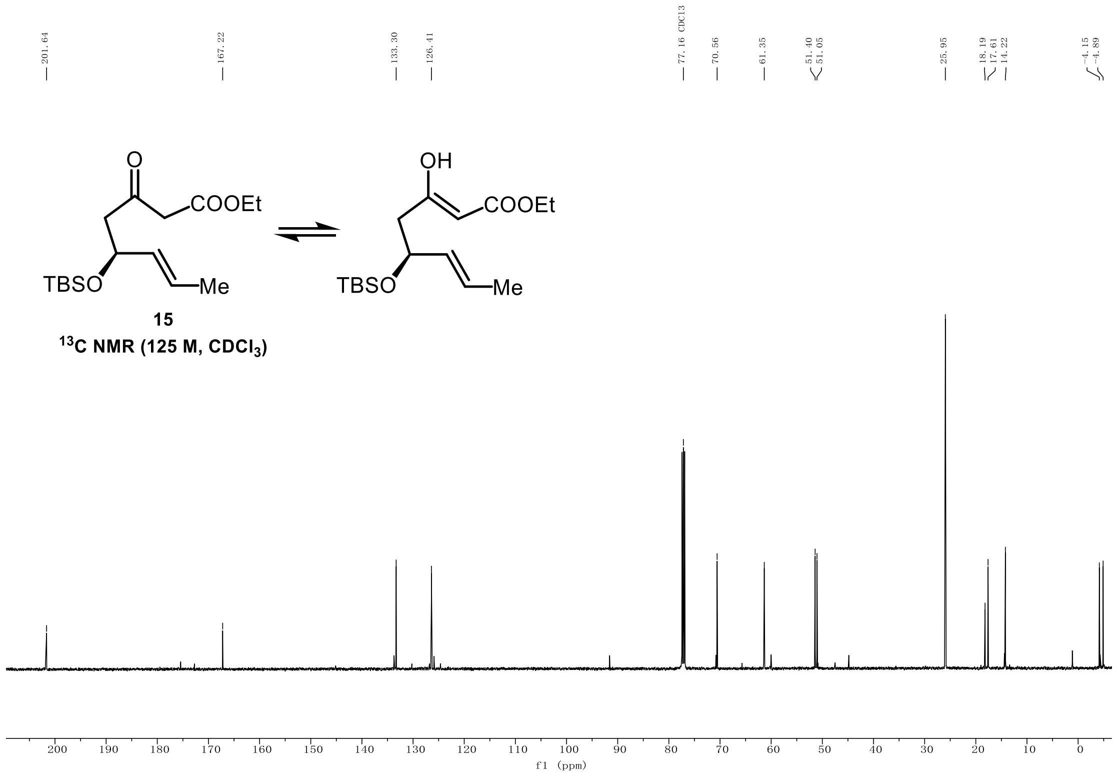

15

^{13}C NMR (125 M, CDCl$_3$)

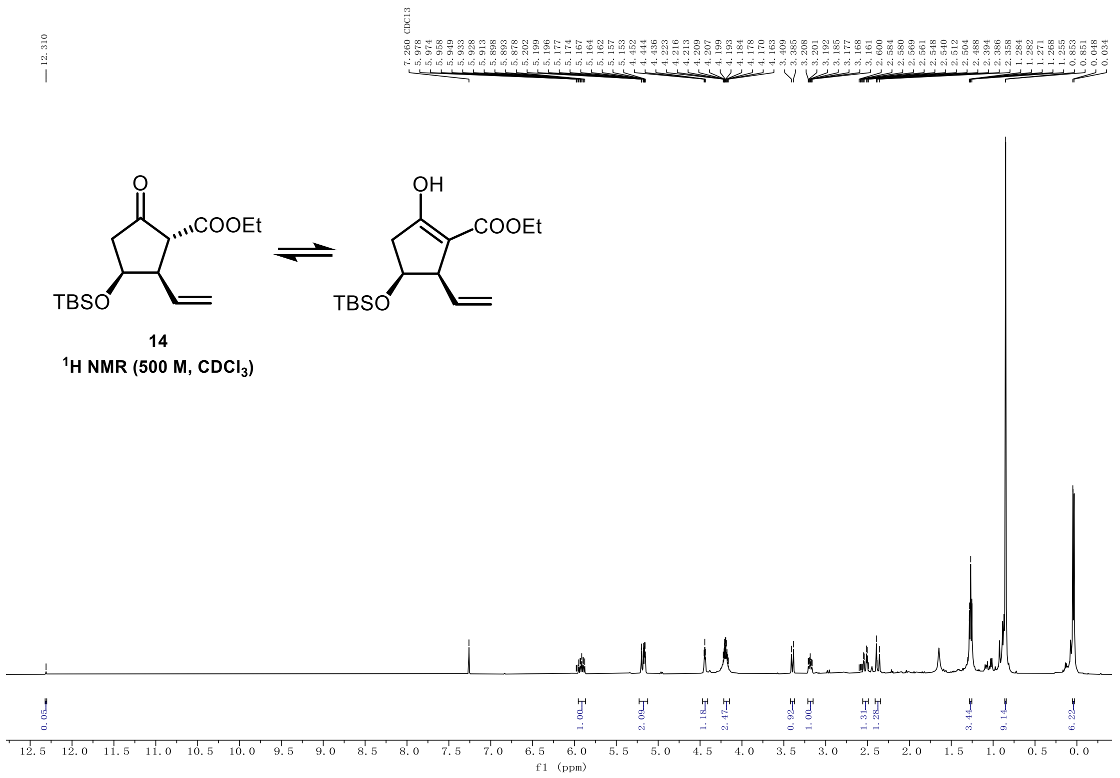

14

^{1}H NMR (500 M, $CDCl_3$)

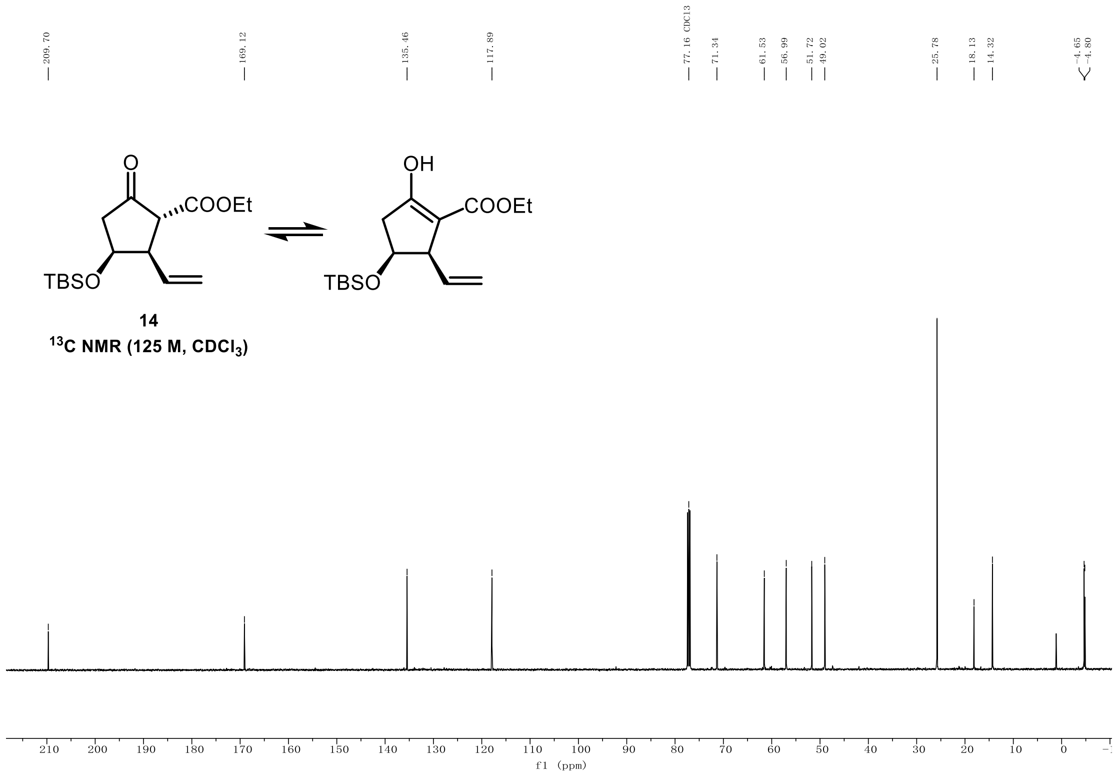

14

^{13}C NMR (125 M, $CDCl_3$)

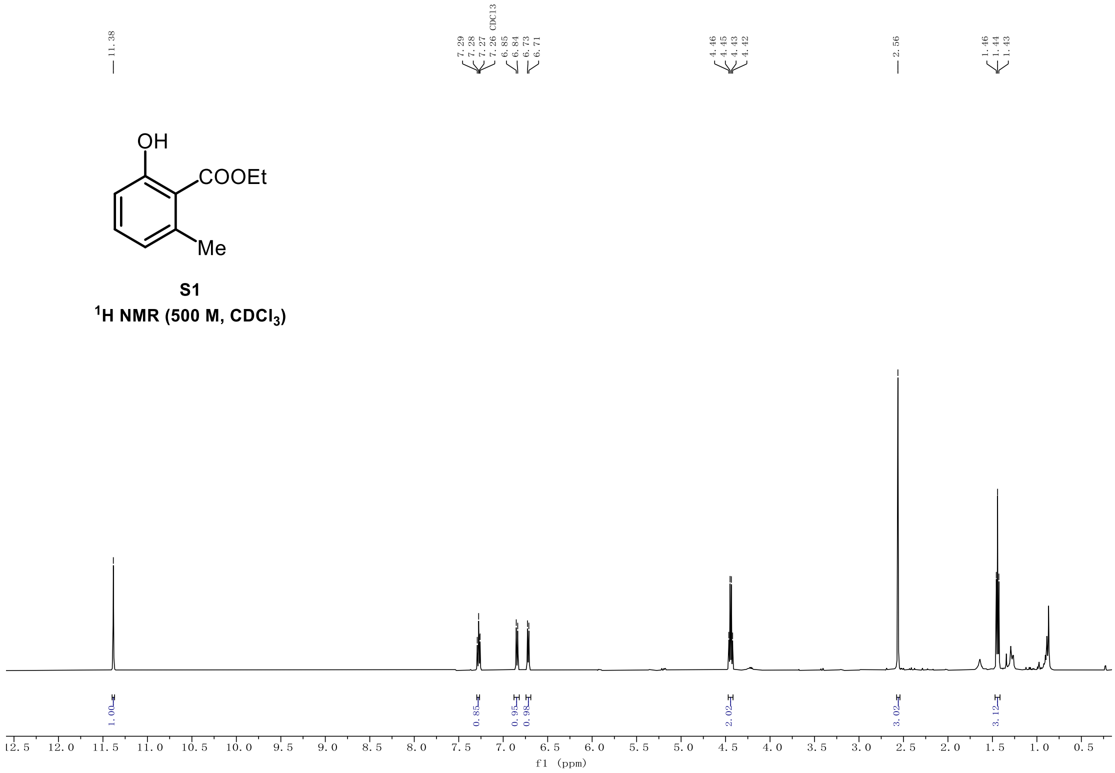

S1
^{1}H NMR (500 M, CDCl$_3$)

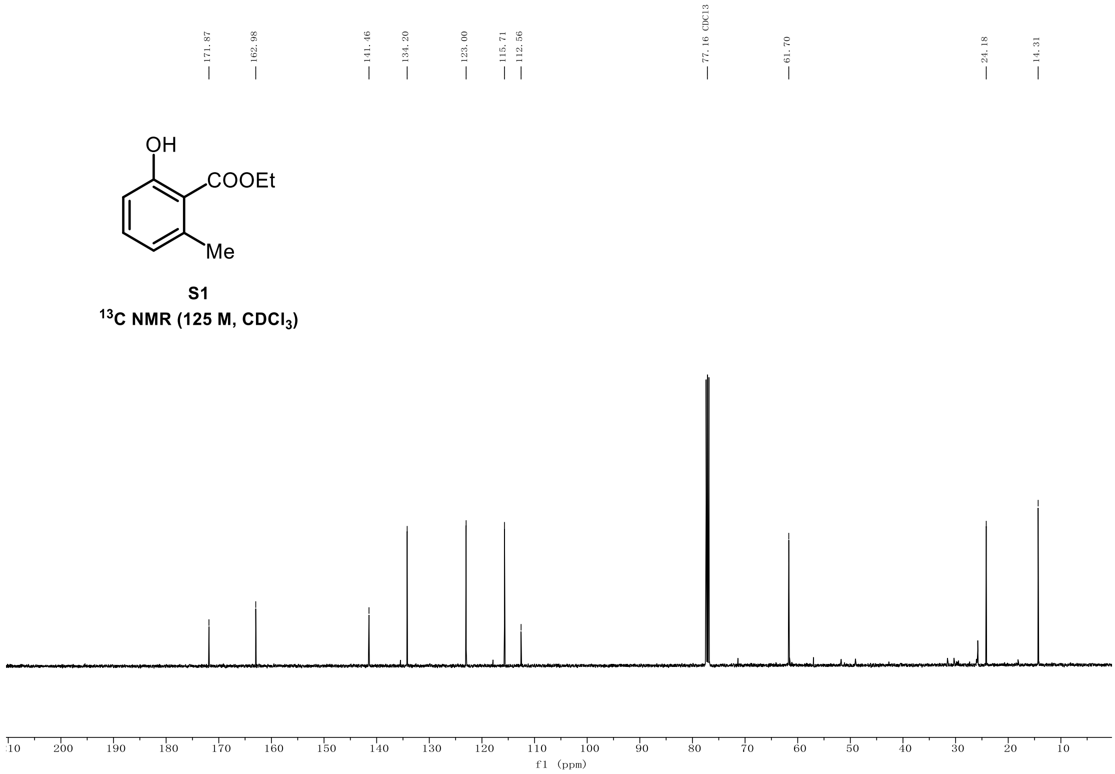

S1
^{13}C NMR (125 M, CDCl$_3$)

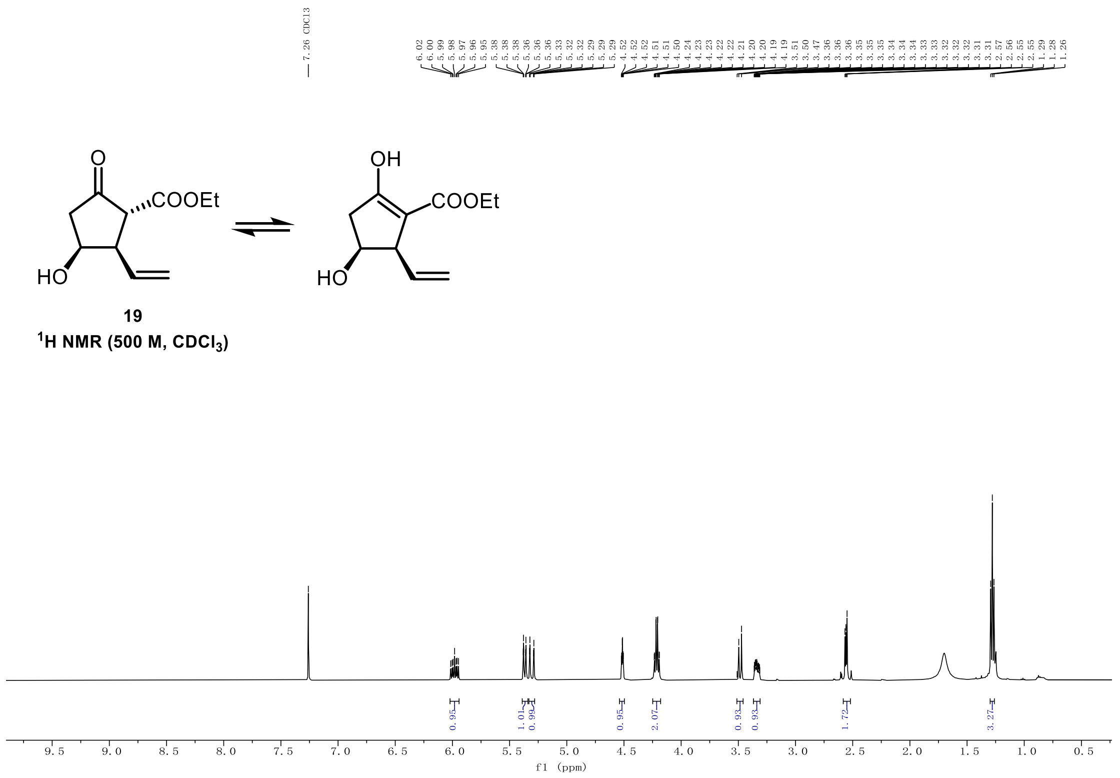

19

^{1}H NMR (500 M, $CDCl_3$)

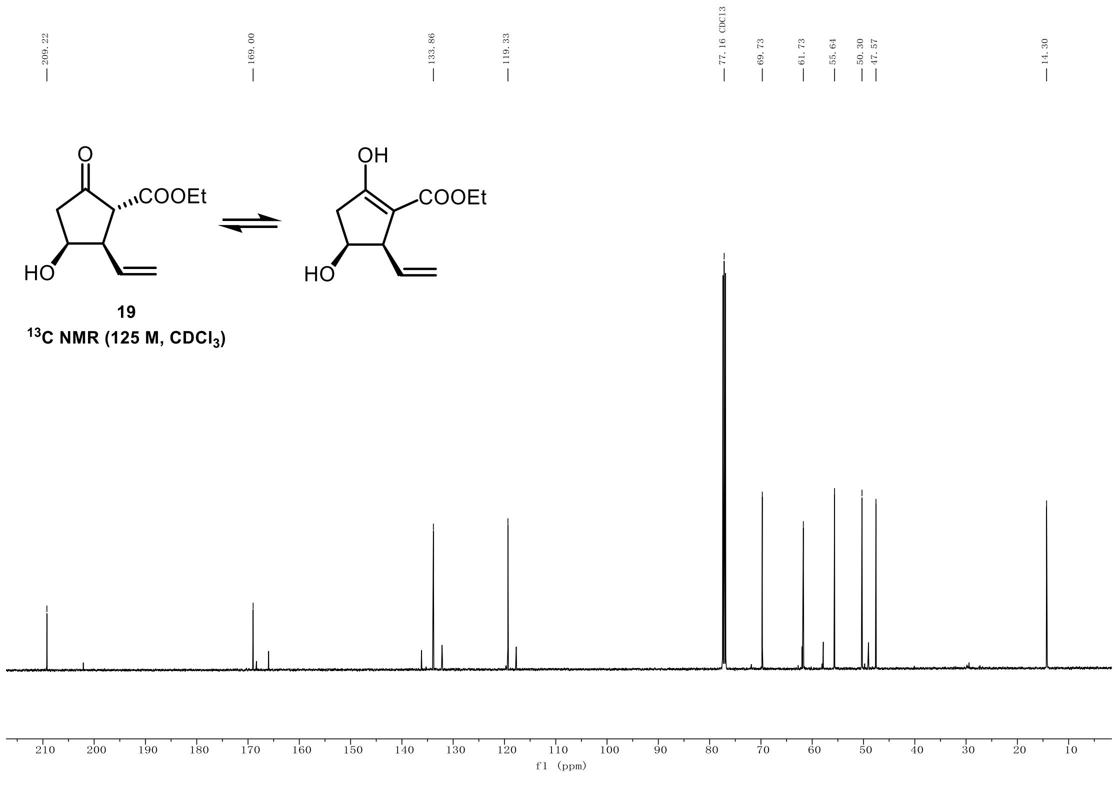

19

^{13}C NMR (125 M, $CDCl_3$)

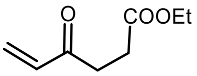

20

^{1}H NMR (500 M, CDCl$_3$)

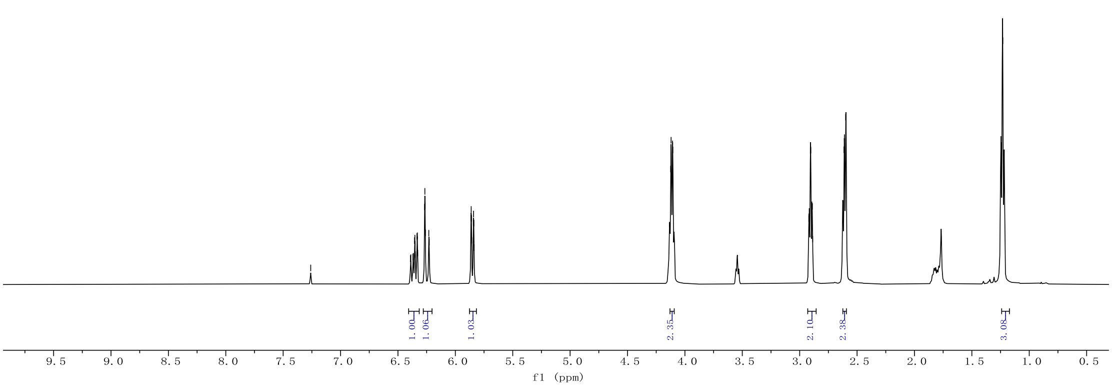

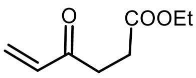

20

^{13}C NMR (125 M, CDCl$_3$)

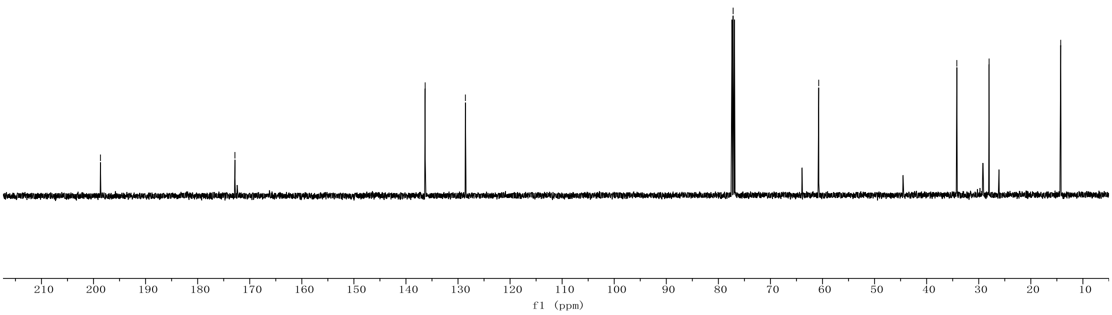

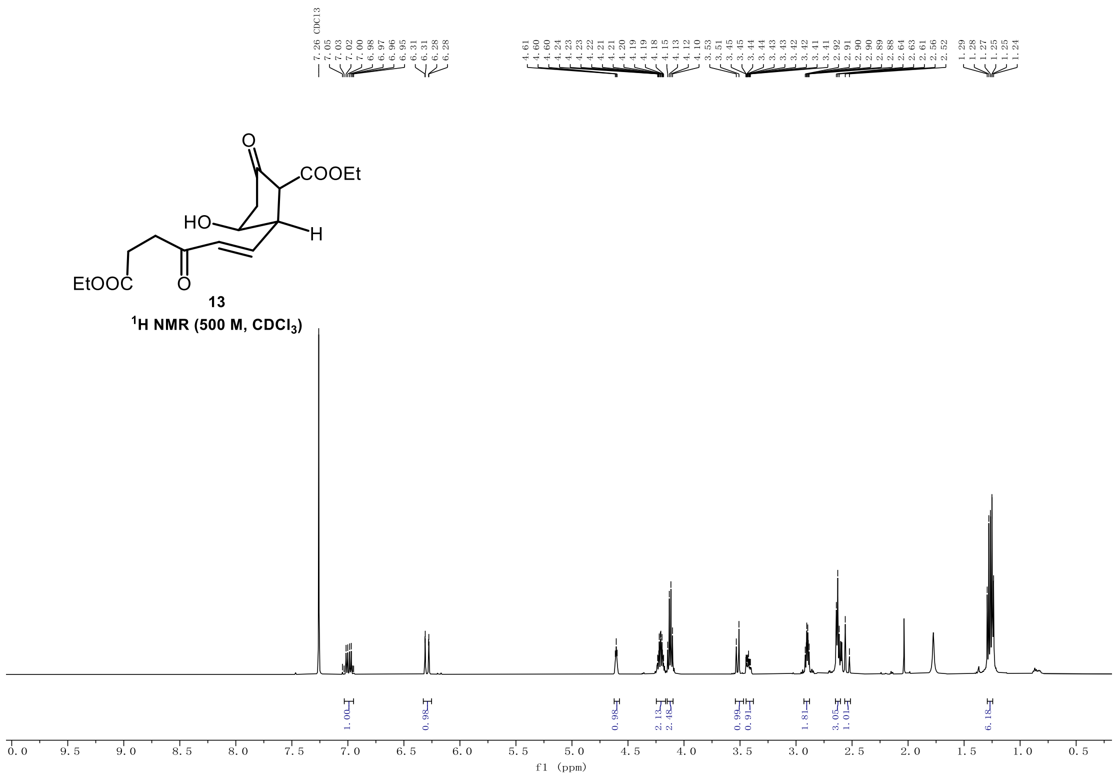
COOEt
HO
H
EtOOC
O
13
1H NMR (500 M, CDCl3)
f1 (ppm)

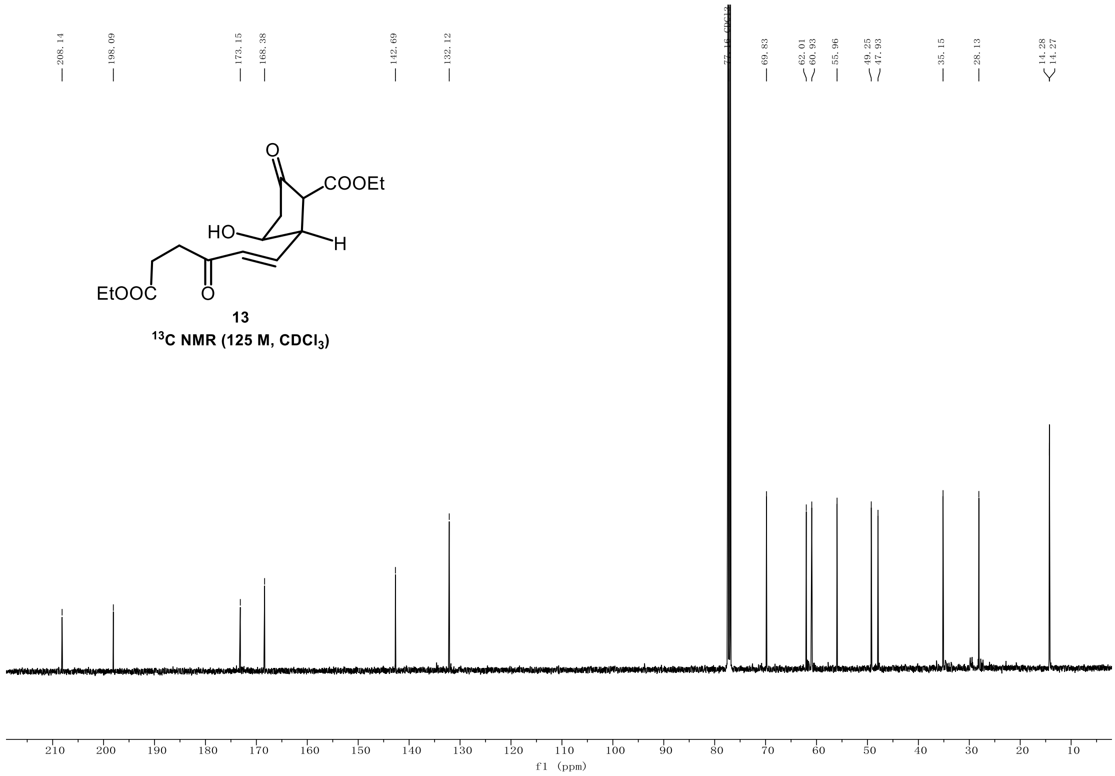
COOEt
HO
H
EtOOC
O
13
13C NMR (125 M, CDCl3)
f1 (ppm)

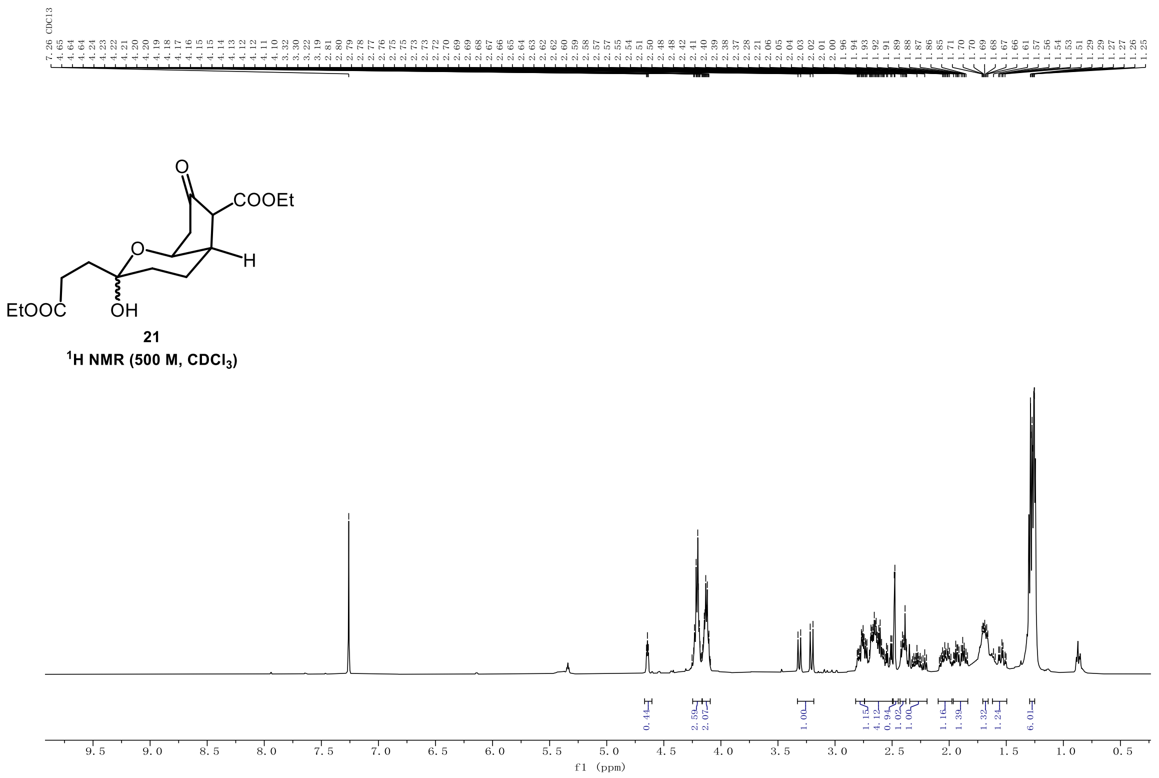

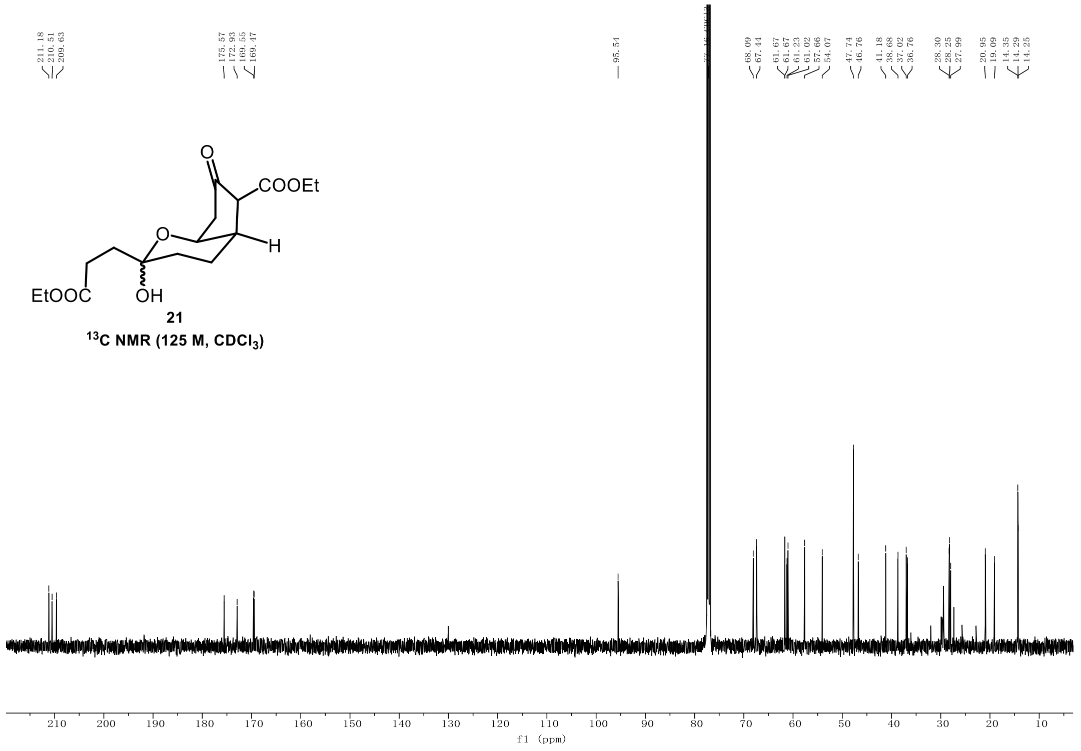

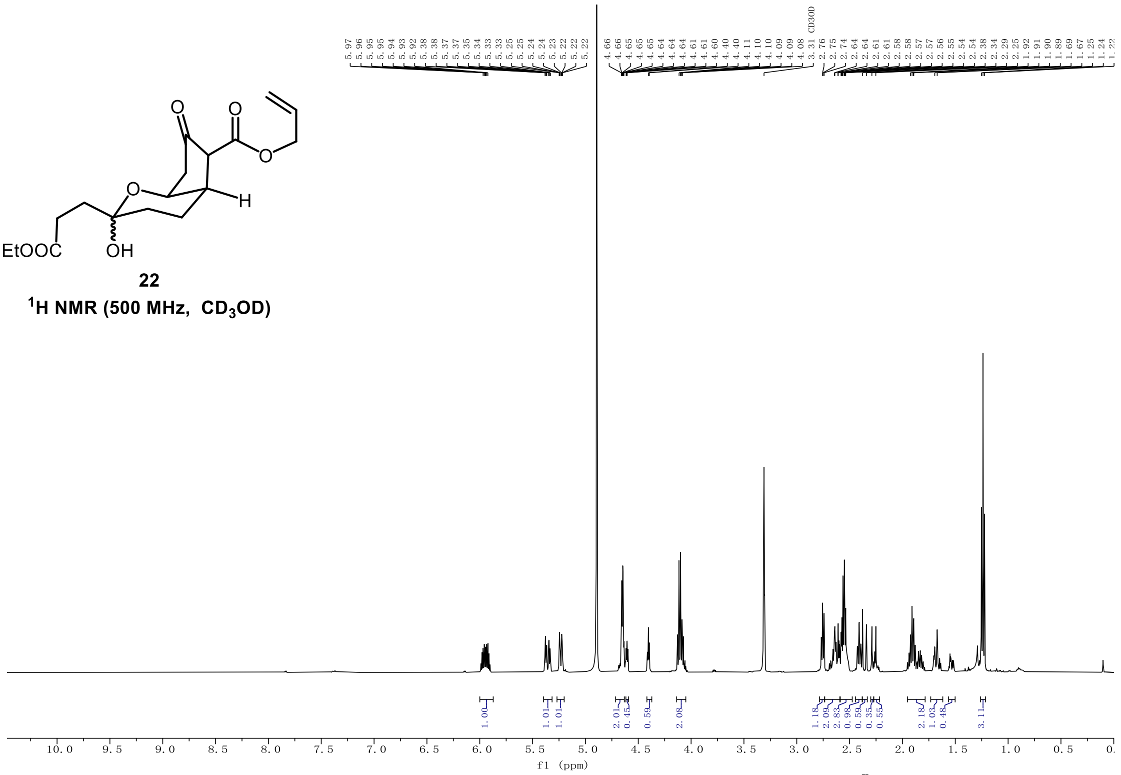

22

^{1}H NMR (500 MHz, CD_3OD)

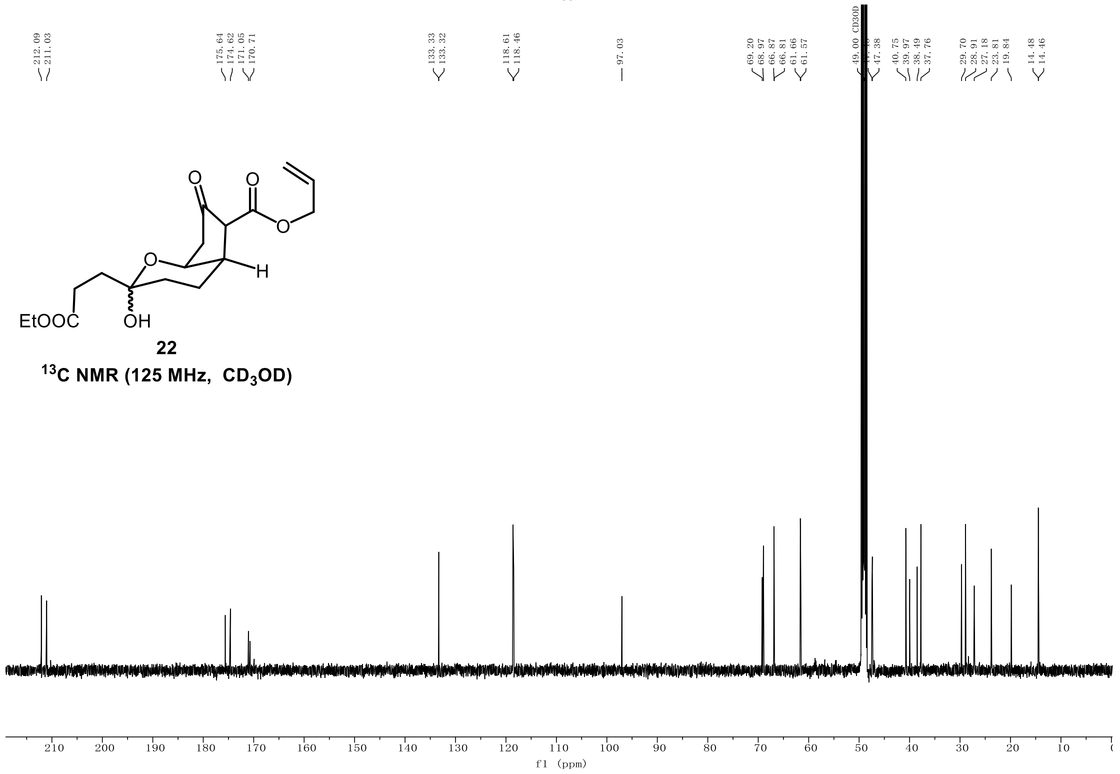

22

^{13}C NMR (125 MHz, CD_3OD)

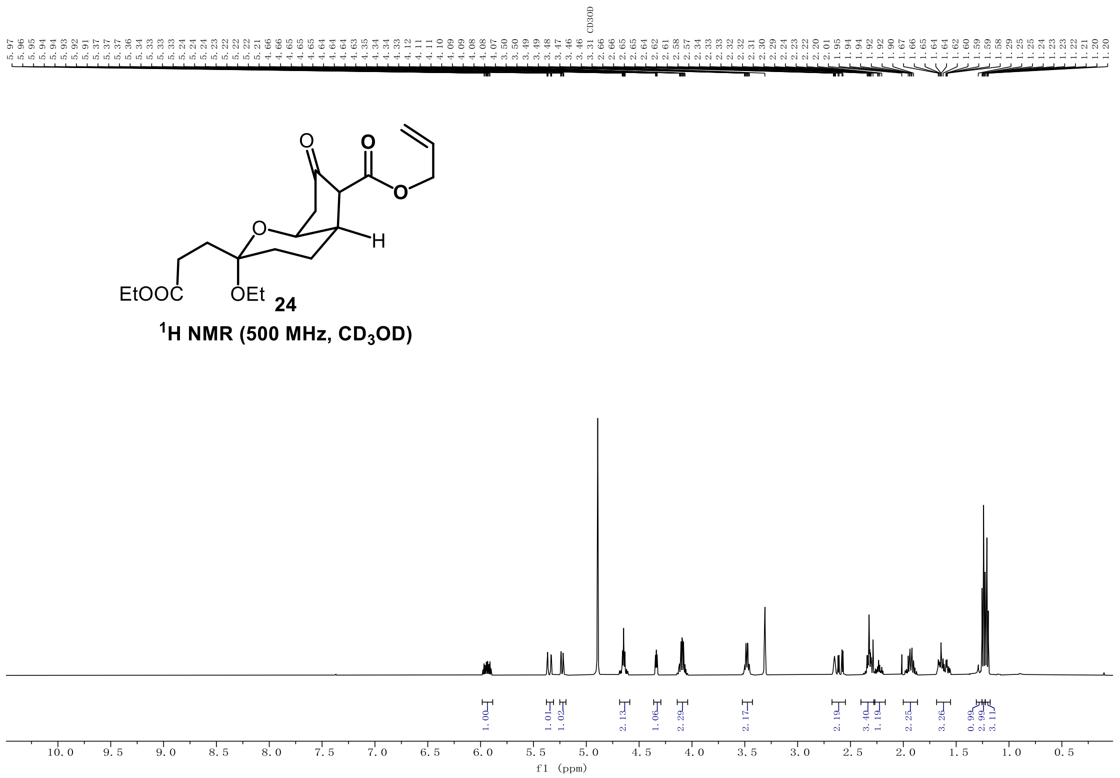
5.97 5.96 5.95 5.94 5.93 5.92 5.91 5.37 5.37 5.36 5.34 5.33 5.33 5.33 5.24 5.24 5.24 5.23 5.22 5.22 5.22 5.21 4.66 4.66 4.65 4.65 4.65 4.64 4.64 4.64 4.63 4.35 4.34 4.34 4.33 4.12 4.11 4.11 4.10 4.09 4.09 4.08 4.08 4.07 3.50 3.50 3.49 3.49 3.48 3.47 3.46 3.46 3.31 CD3OD 2.66 2.66 2.65 2.65 2.64 2.62 2.61 2.58 2.57 2.34 2.33 2.33 2.32 2.32 2.31 2.30 2.29 2.24 2.23 2.22 2.22 2.20 2.01 1.95 1.94 1.94 1.92 1.92 1.90 1.67 1.66 1.65 1.64 1.62 1.60 1.59 1.59 1.58 1.29 1.25 1.25 1.24 1.23 1.23 1.22 1.21 1.20
O
O
O
O
H
EtOOC
OEt
24
1H NMR (500 MHz, CD3OD)
1.00 1.01 1.02 2.13 1.06 2.29 2.17 2.19 3.40 1.19 2.25 3.26 0.99 2.99 3.11
10.0 9.5 9.0 8.5 8.0 7.5 7.0 6.5 6.0 5.5 5.0 4.5 4.0 3.5 3.0 2.5 2.0 1.5 1.0 0.5
f1 (ppm)

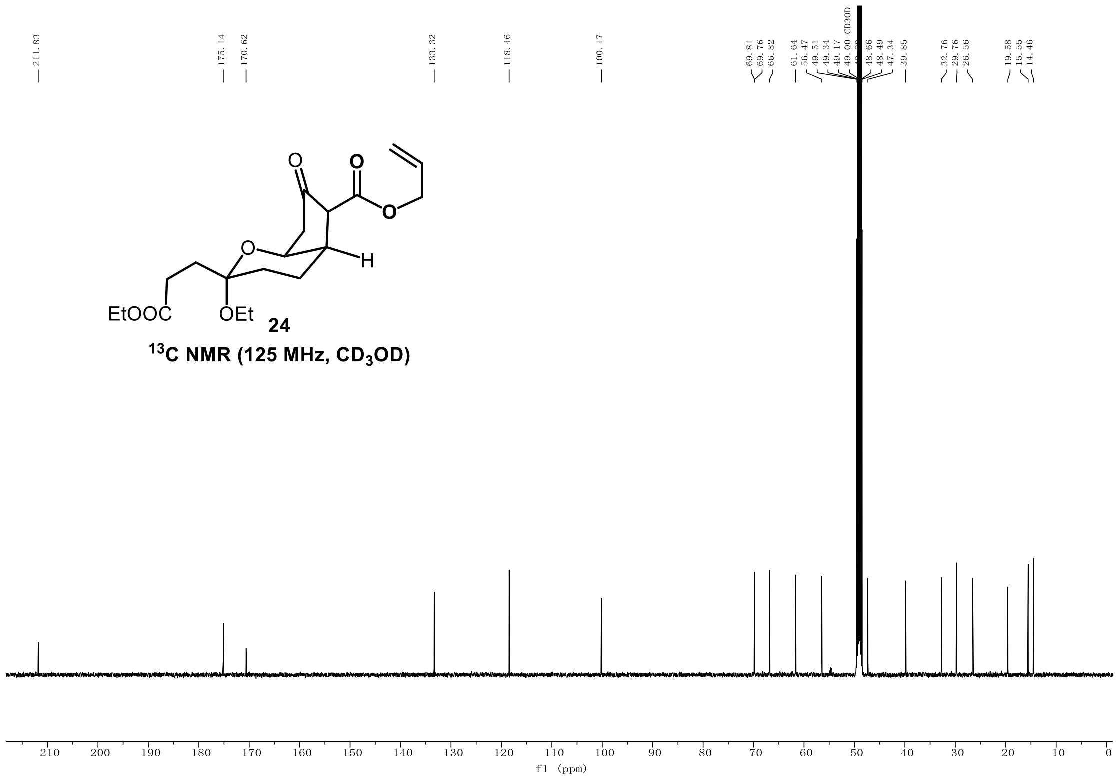
211.83
175.14 170.62
133.32
118.46
100.17
69.81 69.76 66.82
61.64 56.47 49.51 49.34 49.17 49.00 CD3OD 48.66 48.49 47.34
39.85
32.76 29.76 26.56
19.58 15.55 14.46
O
O
O
O
H
EtOOC
OEt
24
13C NMR (125 MHz, CD3OD)
210 200 190 180 170 160 150 140 130 120 110 100 90 80 70 60 50 40 30 20 10 0
f1 (ppm)

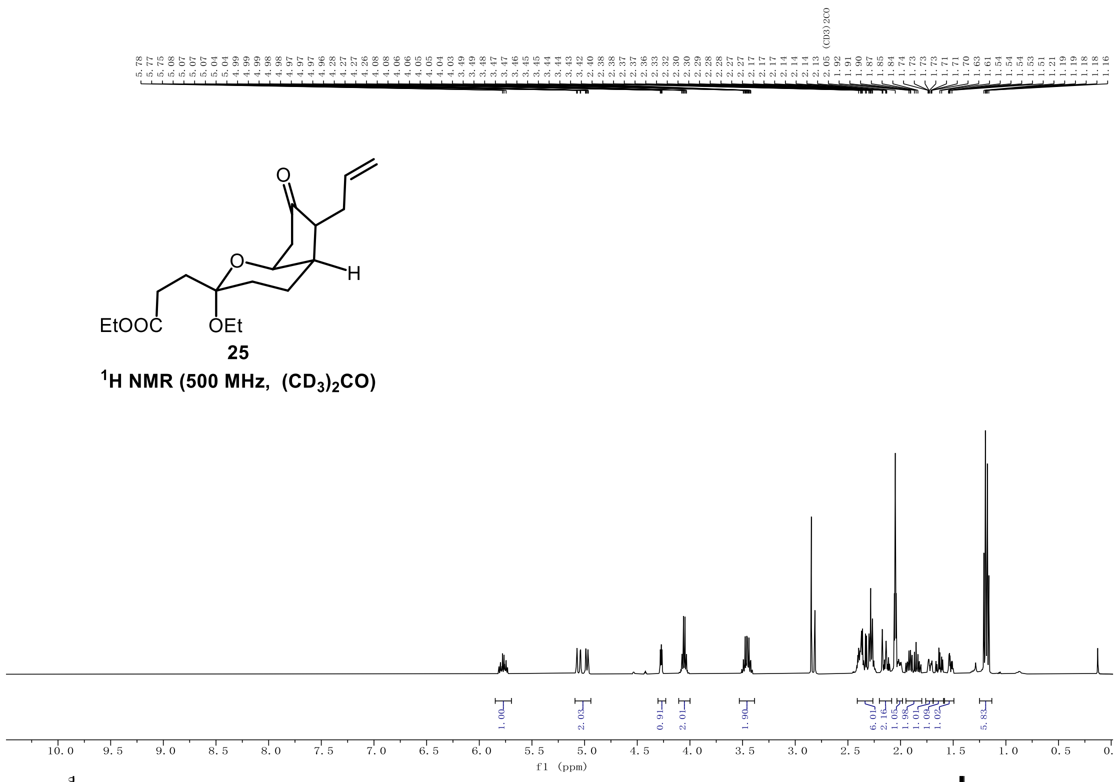

O
H
EtOOC
OEt
25
1H NMR (500 MHz, (CD3)2CO)
(CD3)2CO
1.00
2.03
0.91
2.01
1.90
6.01
2.16
1.05
1.98
1.01
1.04
1.02
5.83
10.0 9.5 9.0 8.5 8.0 7.5 7.0 6.5 6.0 5.5 5.0 4.5 4.0 3.5 3.0 2.5 2.0 1.5 1.0 0.5 0.
f1 (ppm)

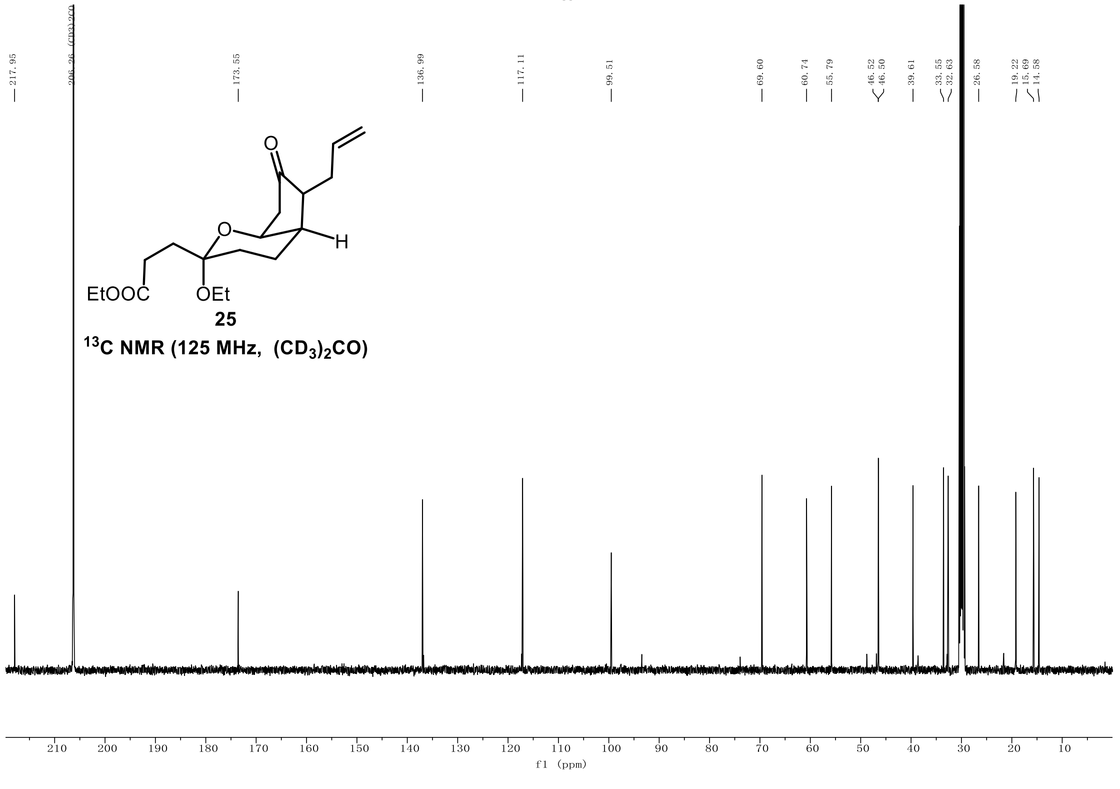

217.95
206.26 (CD3)2CO
173.55
136.99
117.11
99.51
69.60
60.74
55.79
46.52
46.50
39.61
33.55
32.63
26.58
19.22
15.69
14.58
O
H
EtOOC
OEt
25
13C NMR (125 MHz, (CD3)2CO)
210 200 190 180 170 160 150 140 130 120 110 100 90 80 70 60 50 40 30 20 10
f1 (ppm)

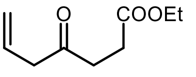

28

¹H NMR (500 MHz, CDCl$_3$)

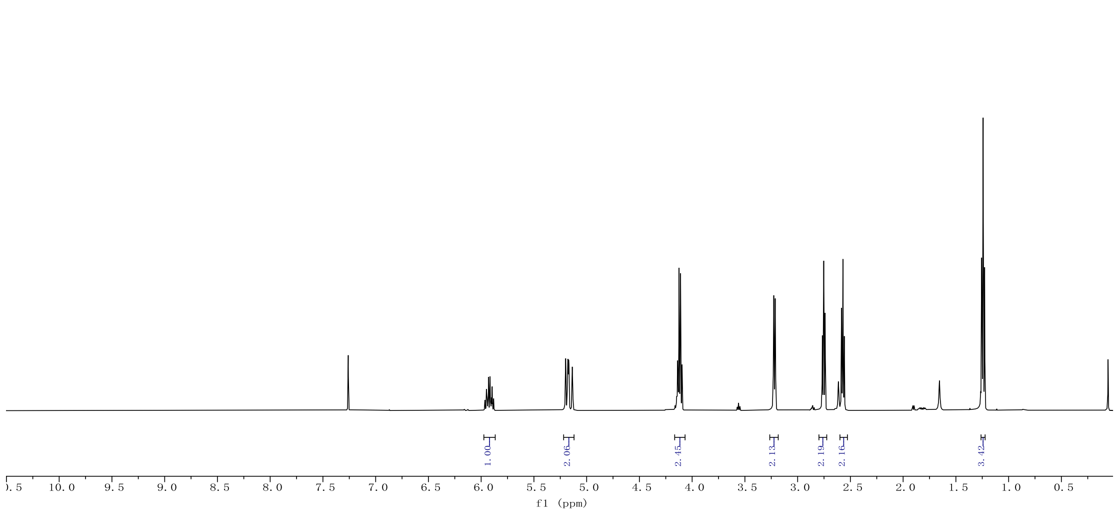

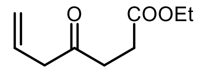

28

¹³C NMR (125 MHz, CDCl$_3$)

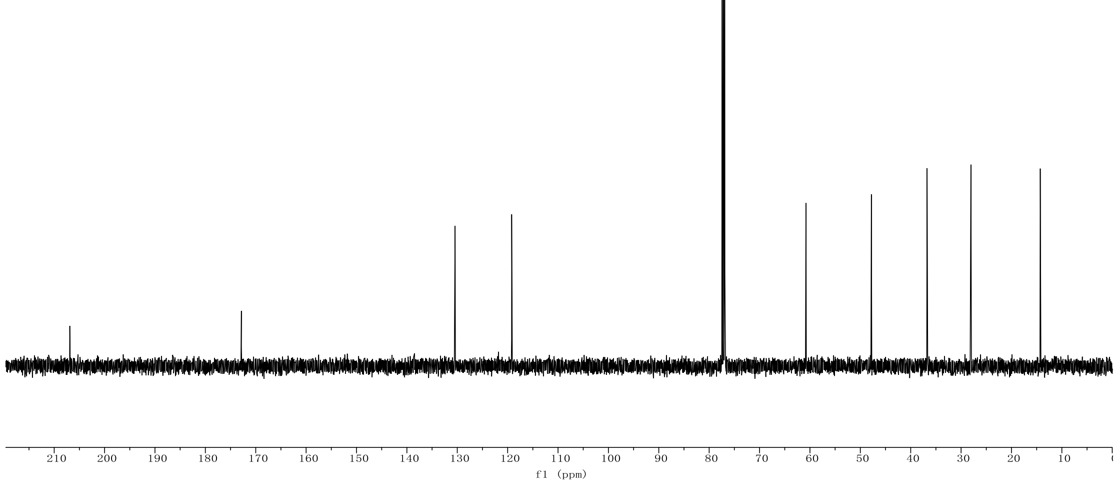

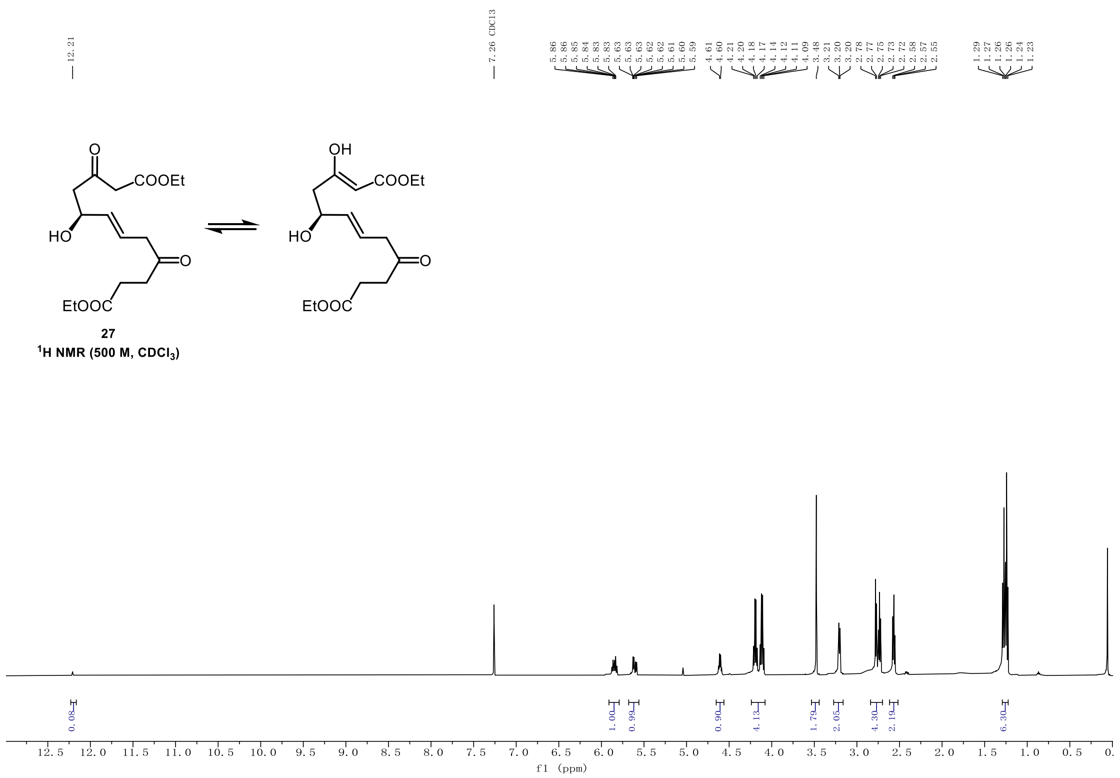

27

^{1}H NMR (500 M, $CDCl_3$)

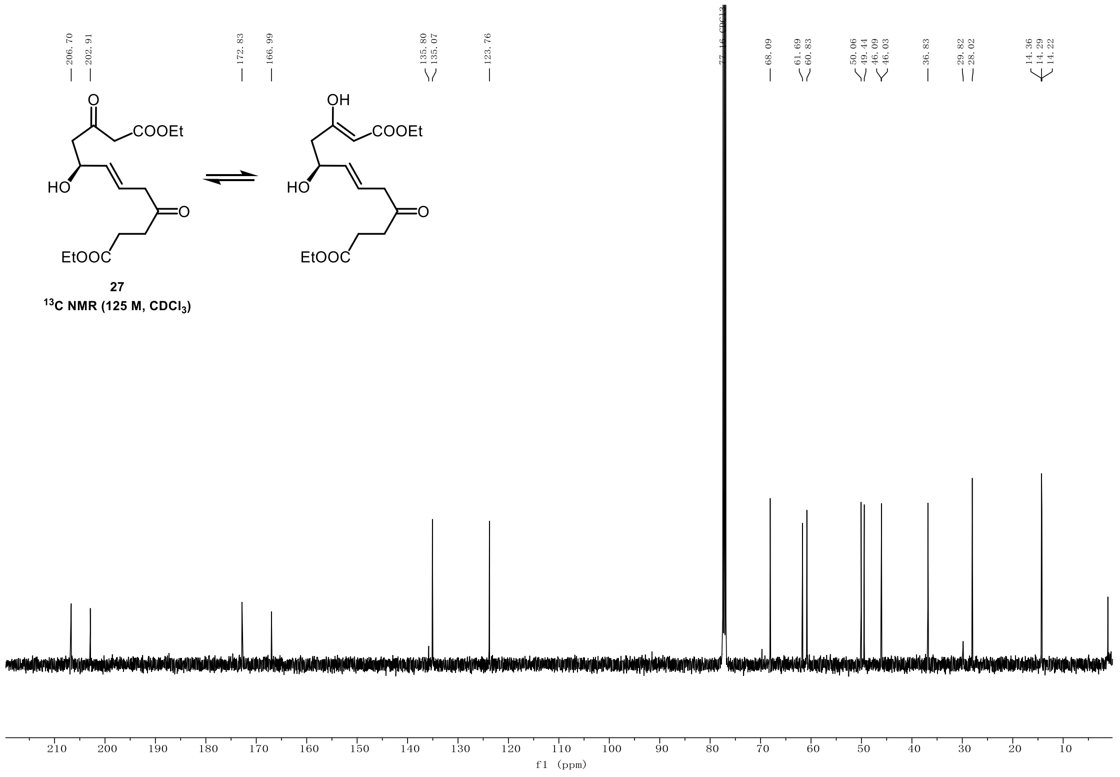

27

^{13}C NMR (125 M, $CDCl_3$)

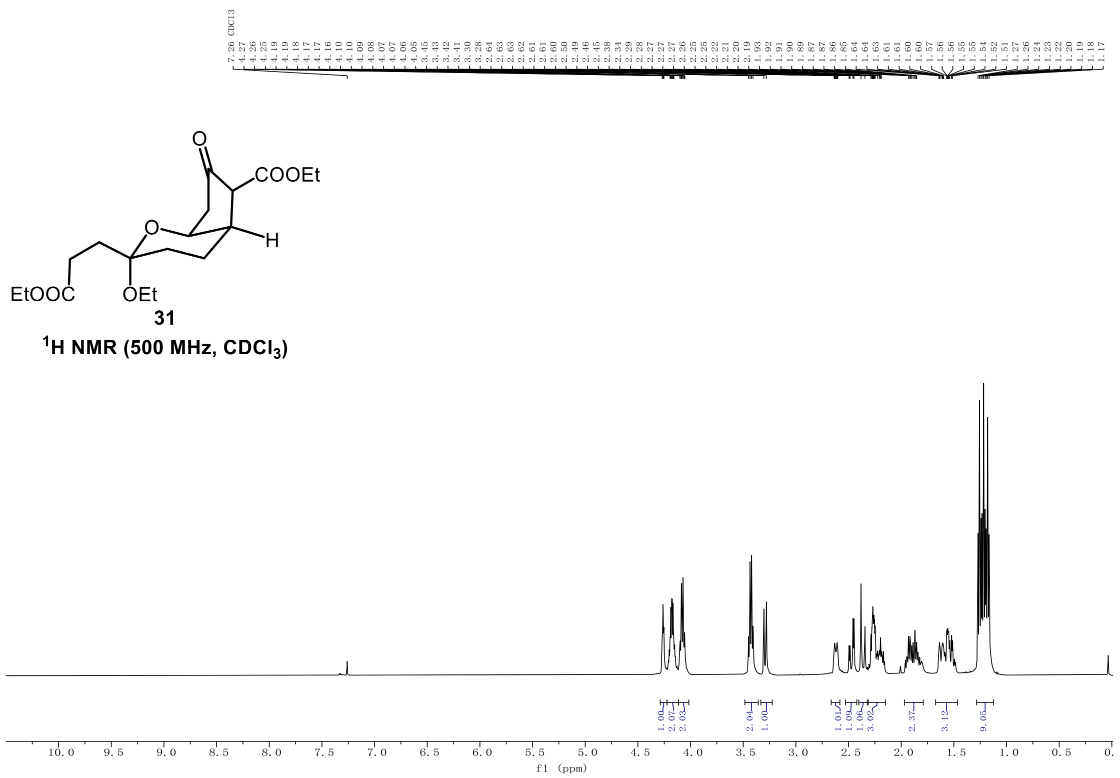

31

^{1}H NMR (500 MHz, $CDCl_3$)

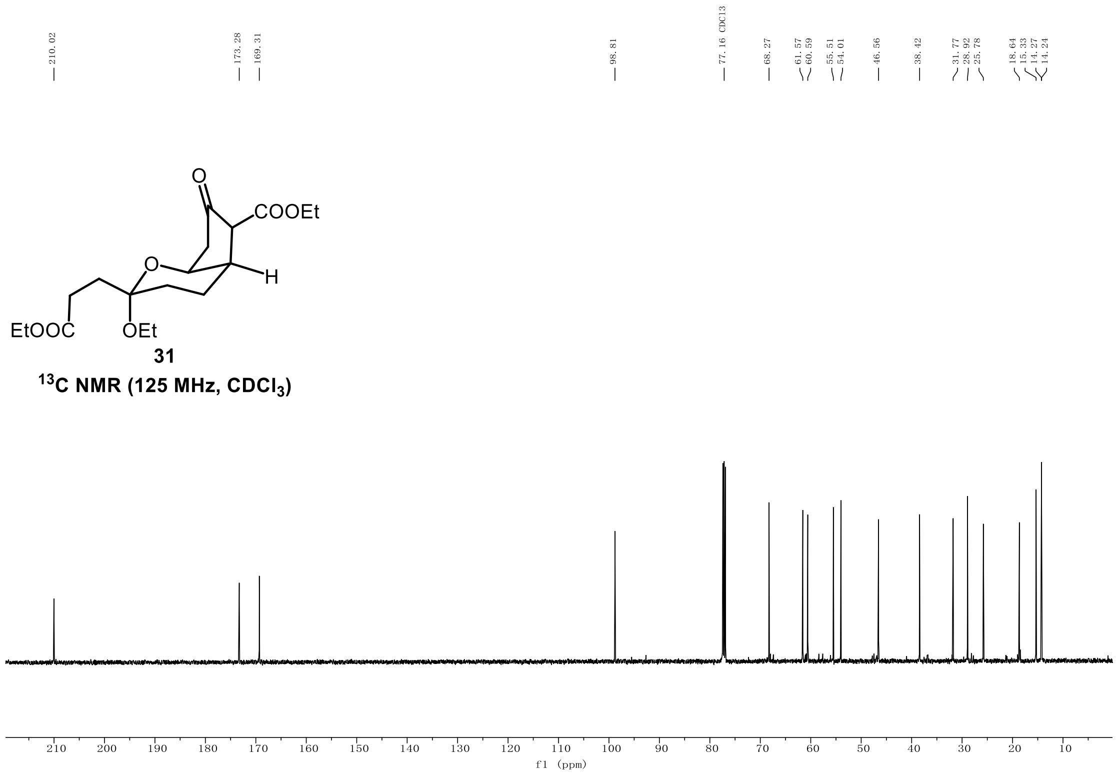

31

^{13}C NMR (125 MHz, $CDCl_3$)

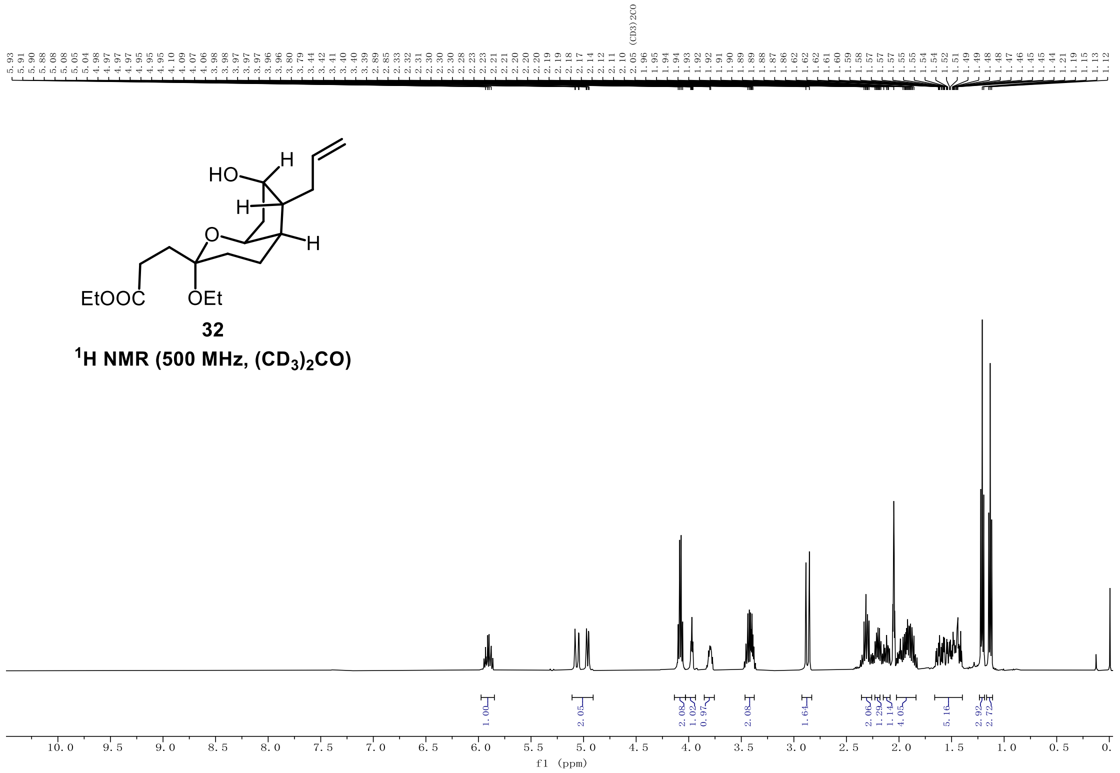

32

^{1}H NMR (500 MHz, $(CD_3)_2CO$)

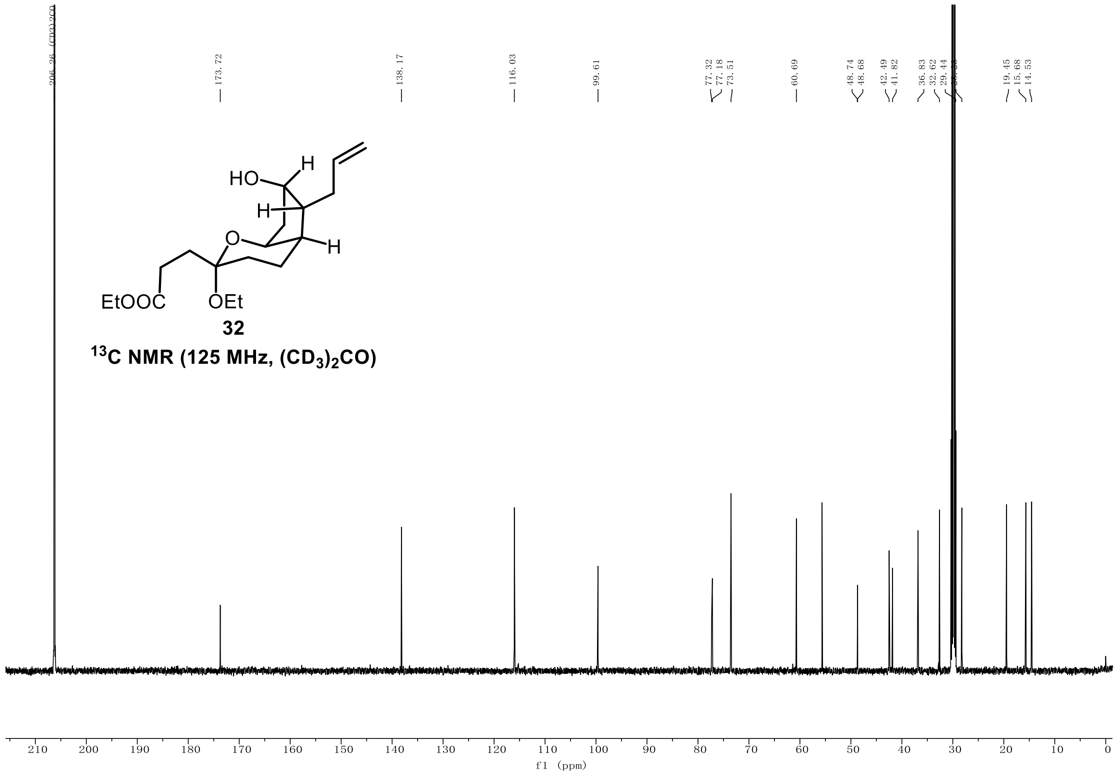

32

^{13}C NMR (125 MHz, $(CD_3)_2CO$)

NOESY spectra of compound 32

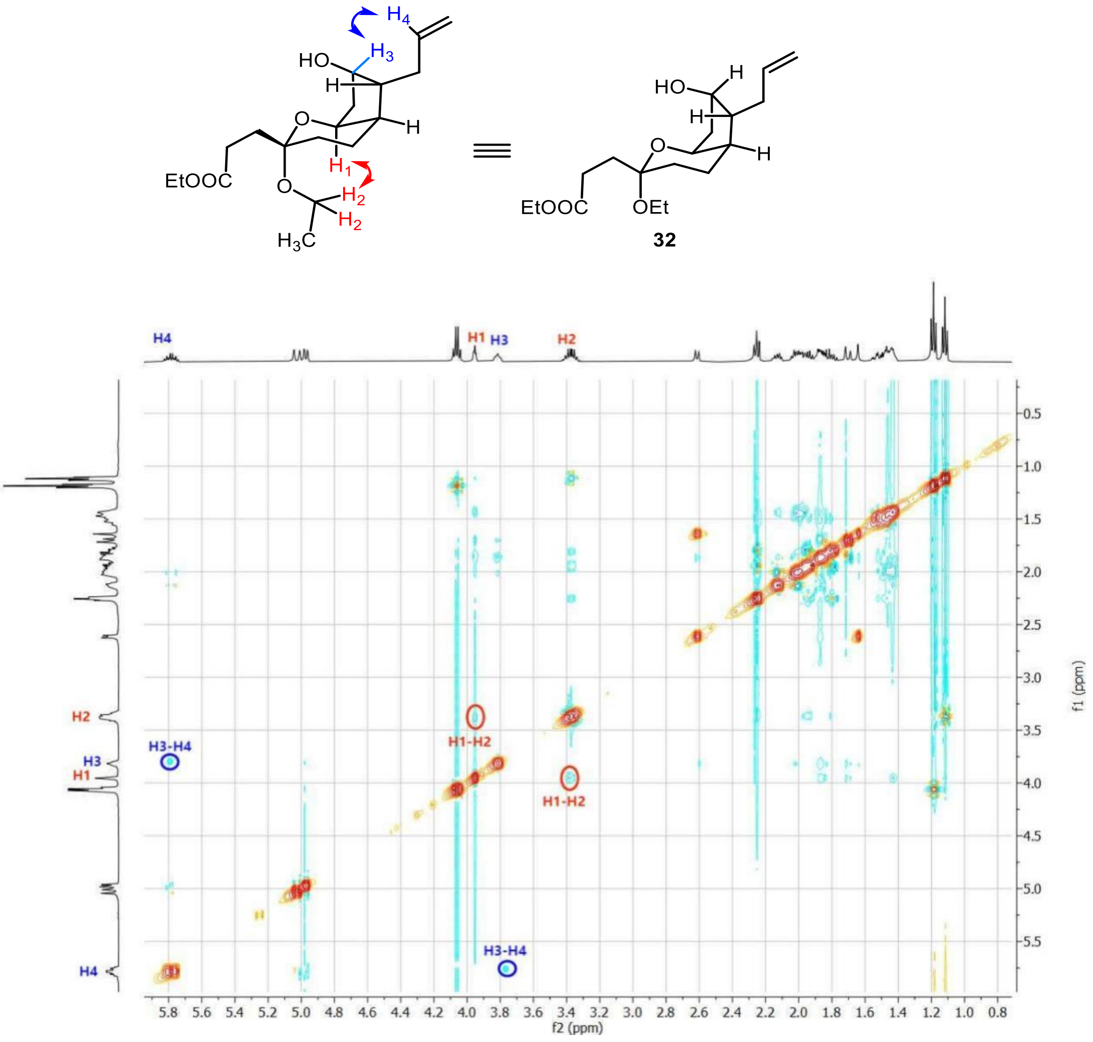

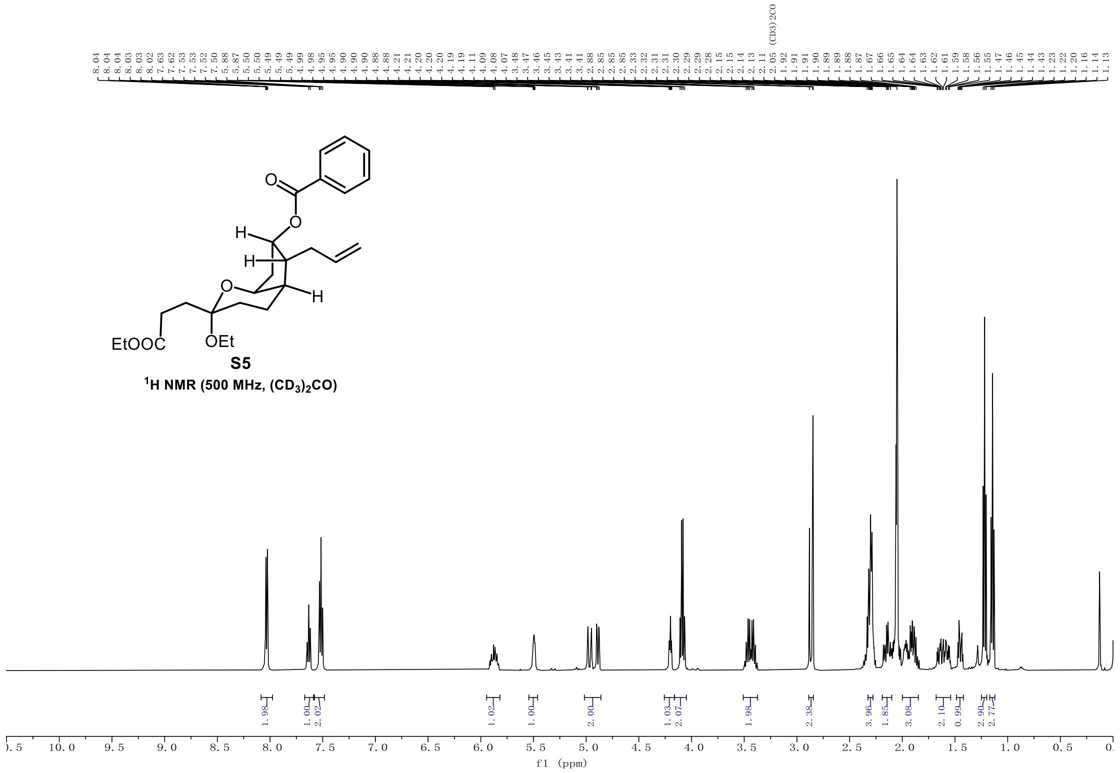
EtOOC
OEt
S5
1H NMR (500 MHz, (CD3)2CO)
f1 (ppm)

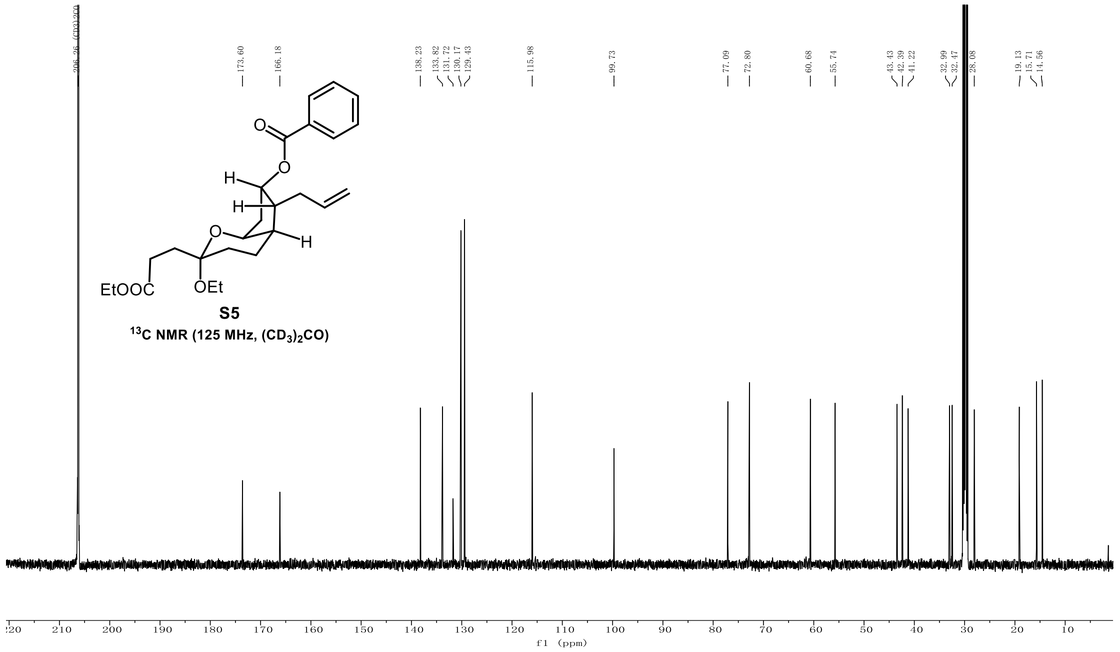
EtOOC
OEt
S5
13C NMR (125 MHz, (CD3)2CO)
f1 (ppm)

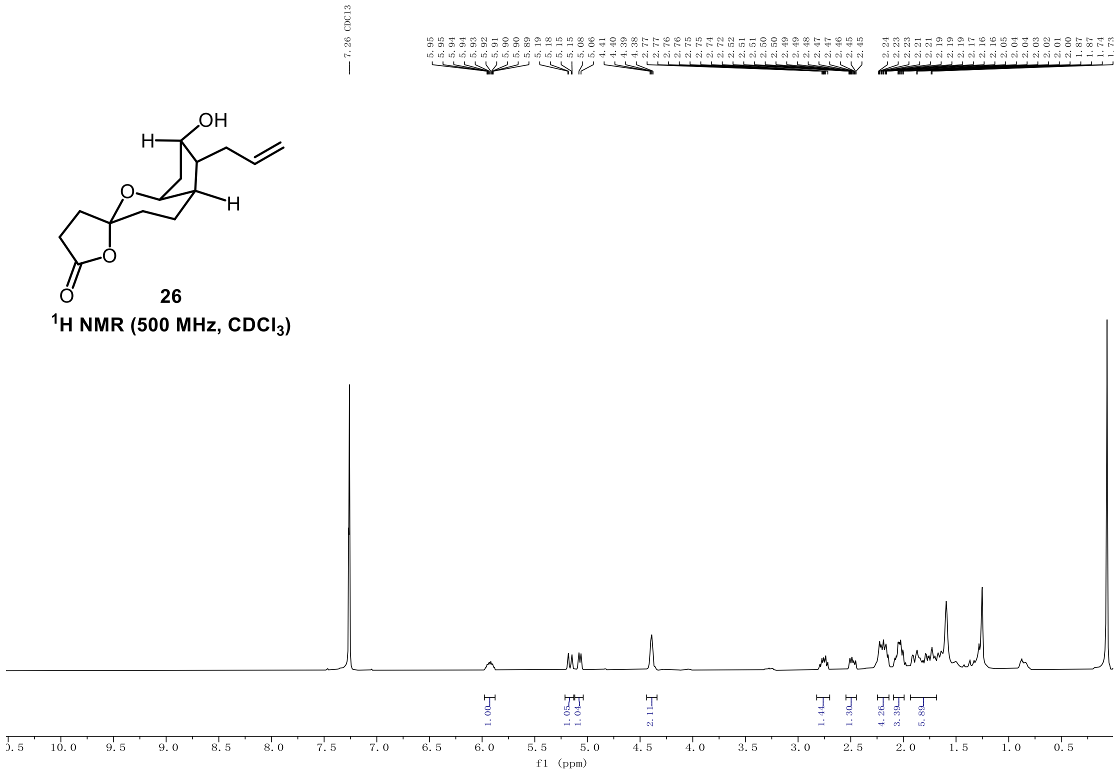

26

^{1}H NMR (500 MHz, $CDCl_3$)

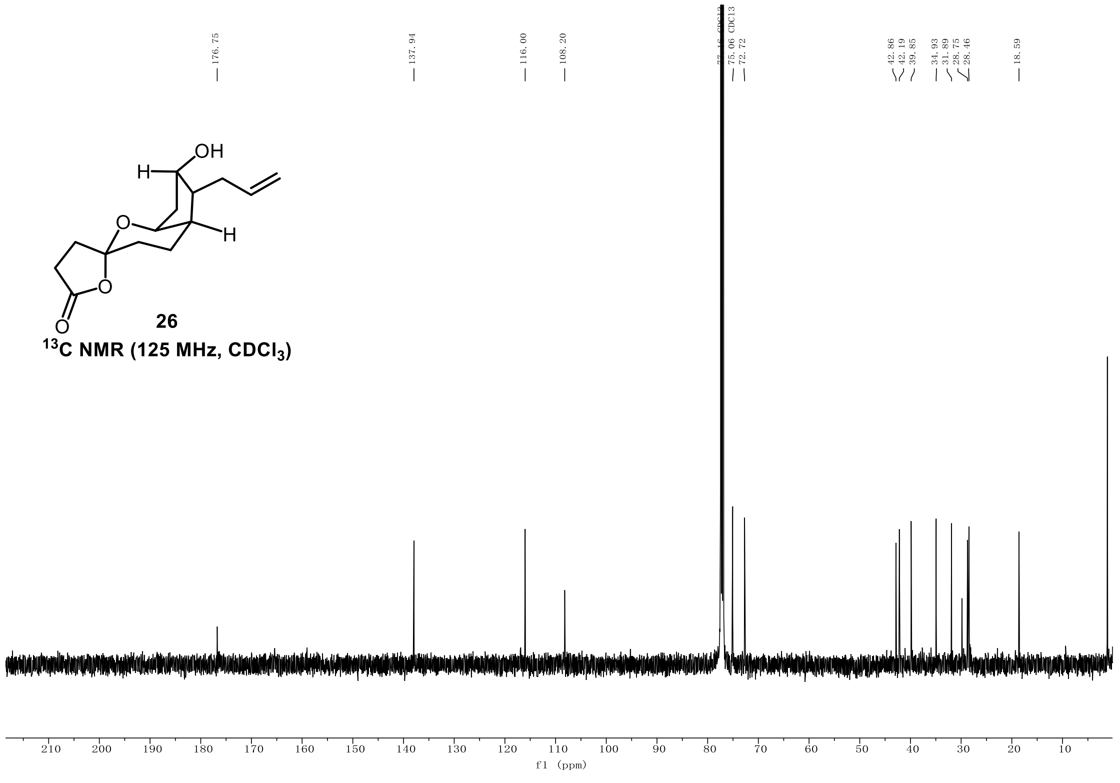

26

^{13}C NMR (125 MHz, $CDCl_3$)

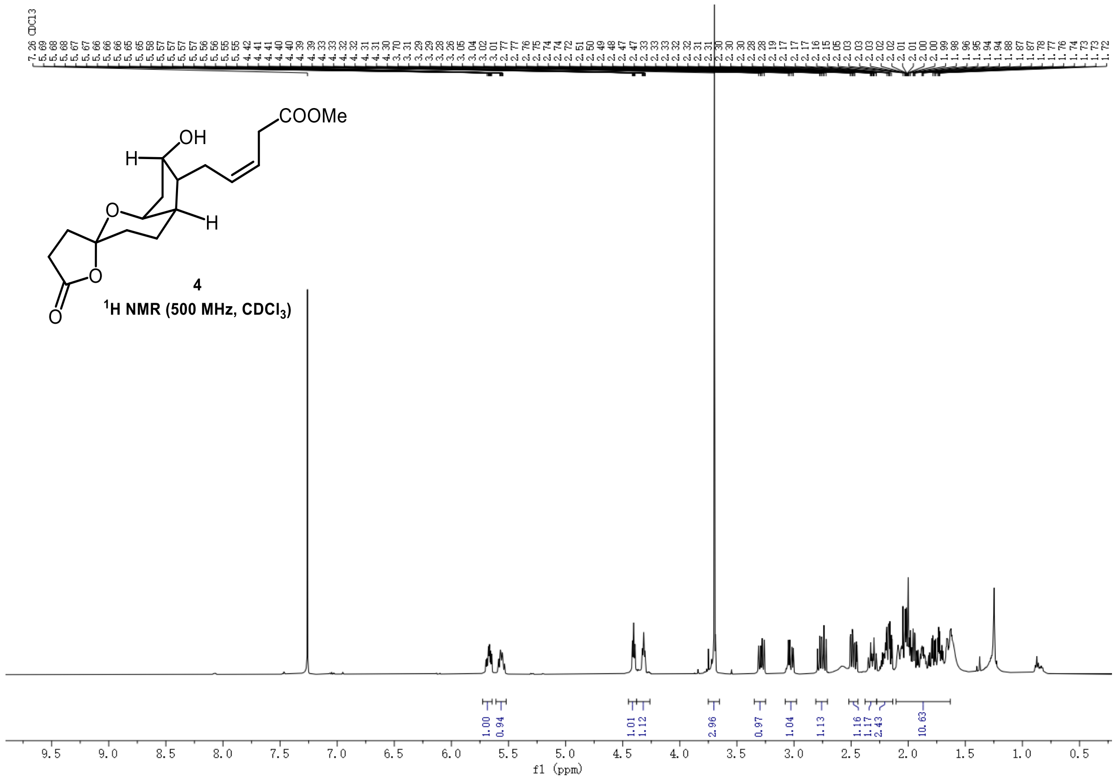
COOMe
OH
H
O
H
O
O
4
1H NMR (500 MHz, CDCl3)
1.00
0.94
1.01
1.12
2.96
0.97
1.04
1.13
1.16
1.17
2.43
10.63
f1 (ppm)

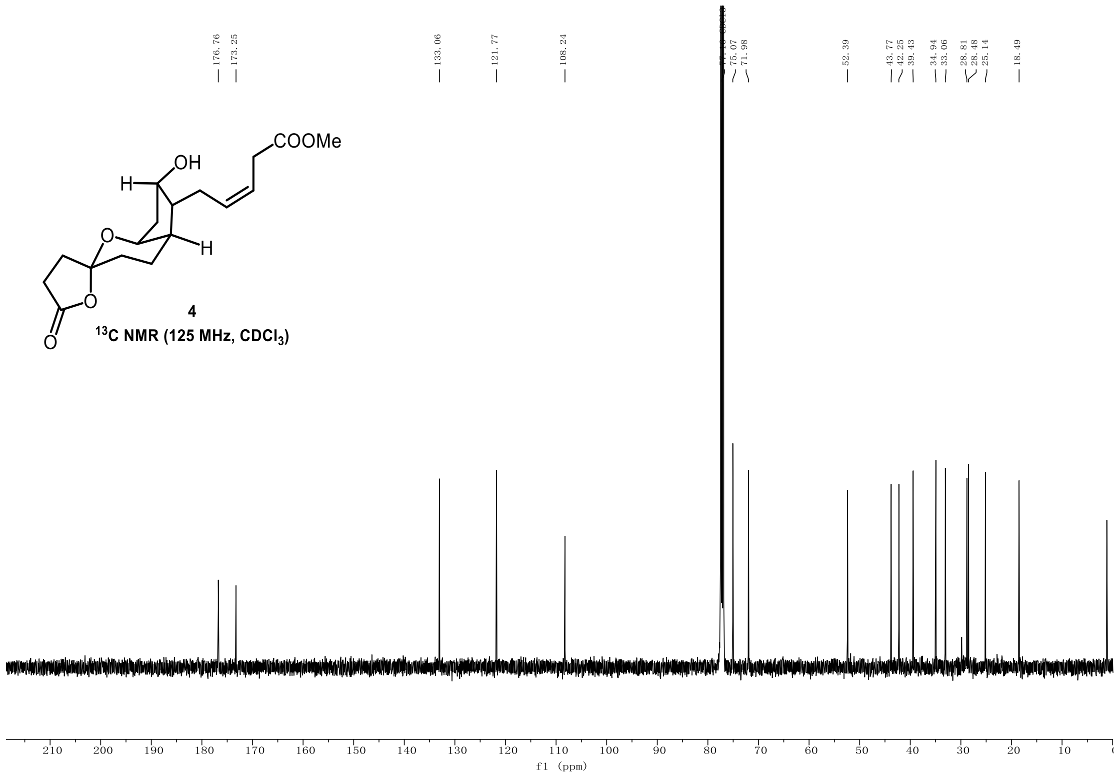
176.76
173.25
133.06
121.77
108.24
75.07
71.98
52.39
43.77
42.25
39.43
34.94
33.06
28.81
28.48
25.14
18.49
COOMe
OH
H
O
H
O
O
4
13C NMR (125 MHz, CDCl3)
f1 (ppm)

5. References

1 Xiao, M.; Shang, Q.; Pu, L.; Wang, Z.; Zhu, Lei.; Yang, Z.; Huang, J. *JACS Au* **2025**, *5*, 1367-1375. doi: 10.1021/jacsau.4c01268.

2 Vasta, J. D.; Higgin, J. J.; Kersteen, E. A.; Raines, R. T. *Bioorgan. Med. Chem*. **2013**, *21*, 3597-3601. doi: org/10.1016/j.bmc.2013.04.057.
